# Recruitment of EB1, a Master Regulator of Microtubule Dynamics, to the Surface of the *Theileria annulata* Schizont

**DOI:** 10.1371/journal.ppat.1003346

**Published:** 2013-05-09

**Authors:** Kerry L. Woods, Romina Theiler, Marcus Mühlemann, Adrian Segiser, Sandra Huber, Hifzur R. Ansari, Arnab Pain, Dirk A. E. Dobbelaere

**Affiliations:** 1 Molecular Pathobiology, Department of Clinical Research and Veterinary Public Health, Vetsuisse Faculty, University of Bern, Bern, Switzerland; 2 Computational Bioscience Research Center (CBRC), Biological and Environmental Sciences and Engineering (BESE) Division, King Abdullah University of Science and Technology (KAUST), Thuwal, Kingdom of Saudi Arabia; Washington University School of Medicine, United States of America

## Abstract

The apicomplexan parasite *Theileria annulata* transforms infected host cells, inducing uncontrolled proliferation and clonal expansion of the parasitized cell population. Shortly after sporozoite entry into the target cell, the surrounding host cell membrane is dissolved and an array of host cell microtubules (MTs) surrounds the parasite, which develops into the transforming schizont. The latter does not egress to invade and transform other cells. Instead, it remains tethered to host cell MTs and, during mitosis and cytokinesis, engages the cell's astral and central spindle MTs to secure its distribution between the two daughter cells. The molecular mechanism by which the schizont recruits and stabilizes host cell MTs is not known. MT minus ends are mostly anchored in the MT organizing center, while the plus ends explore the cellular space, switching constantly between phases of growth and shrinkage (called dynamic instability). Assuming the plus ends of growing MTs provide the first point of contact with the parasite, we focused on the complex protein machinery associated with these structures. We now report how the schizont recruits end-binding protein 1 (EB1), a central component of the MT plus end protein interaction network and key regulator of host cell MT dynamics. Using a range of in vitro experiments, we demonstrate that *T. annulata* p104, a polymorphic antigen expressed on the schizont surface, functions as a genuine EB1-binding protein and can recruit EB1 in the absence of any other parasite proteins. Binding strictly depends on a consensus SxIP motif located in a highly disordered C-terminal region of p104. We further show that parasite interaction with host cell EB1 is cell cycle regulated. This is the first description of a pathogen-encoded protein to interact with EB1 via a bona-fide SxIP motif. Our findings provide important new insight into the mode of interaction between *Theileria* and the host cell cytoskeleton.

## Author Summary

The apicomplexan parasite *Theileria* can reprogram the cell it infects, inducing uncontrolled proliferation and clonal expansion. This is brought about by the schizont, which resides free in the host cell cytoplasm. As the schizont never leaves the cell to infect other cells, it can only persist provided it is distributed over the two daughter cells each time the host cell divides. This is achieved by interacting dynamically with microtubules (MTs) that form part of the host cell mitotic apparatus. How MTs are recruited to the schizont surface is not known. MTs are highly dynamic, undergoing continuous cycles of growth and shrinkage that is regulated to a large extent by an array of proteins, called +TIPs, that associate with the free plus-ends of MTs. End-binding protein 1 (EB1) is a master regulator and central adaptor that mediates MT plus-end tracking of potentially all other +TIPs. We established that a schizont surface protein, p104, provides a docking site for EB1, which critically depends on a consensus SxIP motif, present in p104. These finding provides important new insight into the complex interaction of the transforming schizont with host cell MTs. To our knowledge, p104 is the first pathogen-derived protein identified so far to join the SxIP family of EB1-binding proteins.

## Introduction

The tick-borne Apicomplexan parasites, *Theileria annulata* and *Theileria parva*, are the causative agents of lymphoproliferative diseases of cattle, tropical Theileriosis and East Coast fever, that cause significant economic losses in large parts of Asia and Africa. *T. annulata* predominantly infects macrophages/monocytes and B-cells, while *T. parva* infects predominantly T-cells and B-cells. Both species possess the unique capacity of transforming their host cells, inducing uncontrolled proliferation and resistance to apoptosis (reviewed in [1], [2])

Like other Apicomplexan parasites (such as *Toxoplasma*. and *Plasmodium spp*.), the life cycle of *Theileria* is complex and involves several morphologically different stages. Sporozoite entry in the target cells is a rapid process and within 15–30 minutes of invasion the infective sporozoite eliminates the enclosing host cell membrane upon which it associates with host cell MTs. Free in the cytoplasm, the parasite then differentiates into a multinucleated syncytium called a schizont [3]. Not restricted by the confines of a parasitophorous vacuole, the transforming schizont is in a perfect position to interfere with host cell signaling pathways that regulate cell proliferation and survival [1]. The schizont is strictly intracellular and depends entirely on its interaction with host cell MTs to ensure its persistence within the host cell - and thus maintenance of the transformed phenotype. By associating with the mitotic apparatus during mitosis and cytokinesis, the parasite secures the equal distribution of the schizont between the two new daughter cells [4]. This process involves the recruitment and stable association of de novo synthesized astral and central spindle MTs with the schizont surface [5]. We found that the mitotic kinase polo-like kinase 1 (Plk1) is recruited to the schizont surface in a cell cycle-dependent manner, and that association of the schizont with the central spindle, but not astral MTs, depends upon Plk1 activity. The distribution of the schizont between the two daughter cells during host cell cytokinesis is dependent on this association and by preventing the interaction with both astral and central spindle MTs proper segregation between the two daughter cells is disrupted [5].

The molecular mechanisms by which MTs are recruited and stabilized at the parasite surface are largely unknown. Proteins expressed on the surface of the schizont, or those secreted into the cytoplasm, qualify as good candidates for mediating host-parasite interactions, and yet to date only a few schizont surface proteins have been characterized in detail. gp34, a GPI-anchored schizont surface protein has been implicated in host-parasite interactions during host cell division [6]. It was proposed that TaSP, a proline-rich immunodominant *T. annulata* surface protein, can bind host cell α-tubulin and interact with the host cell microtubular network, but the molecular basis for these interactions is unclear [7].

MTs are involved in many cellular processes including cell differentiation, vesicle transport and cell division, and interact with various intracellular structures, including the actin cytoskeleton, the cell cortex and kinetochores. MTs are polarized, possessing minus ends that are usually stabilized and embedded in MT-organizing centers (MTOCs), and plus ends that extend into the cytoplasm and switch between periods of growth and shrinkage in a process termed 'dynamic instability [8]. MT dynamics are regulated to a large extent by MT associated proteins (MAPs), an important subset of which is the plus end tracking proteins (+TIPs). +TIPs constitute a structurally diverse family of MAPs that localize to the growing plus ends of MTs and play important roles in the regulation of MT growth and stability (reviewed in [9]). Since the description of the first +TIP, cytoplasmic linker protein CLIP-170, more than 12 years ago [10], the +TIP family has continuously expanded. They contribute to the regulation of mitosis, maintenance of cell polarity and positioning of organelles.

A network of +TIPs regulates different aspects of MT dynamics; XMAP215 promotes MT growth by catalyzing the addition of tubulin subunits to the growing MT plus end [11], while end-binding proteins (EBs) promote MT growth by suppressing catastrophes [12]). CLIP-170 promotes MT rescue [13], CLASP promotes MT stability [14] and the kinesin MCAK (XKCM1) has MT depolymerizing activity [15], [16]. By forming dynamic interaction networks with one another and with MTs, +TIPs cooperate to tightly control the rates of MT growth and shrinkage and finely tune MT dynamics that is so crucial for many functions, including the faithful segregation of chromosomes and successful cell division (for reviews, see [17], [18], [19]).

EBs are evolutionarily conserved in mammals, plants, fungi and other lower eukaryotes, and take a central position at the hub of the +TIP interaction network. EB1 was originally identified as a binding partner of adenomatous polyposis coli (APC) tumor suppressor protein [20]. EBs can bind directly to growing MT plus ends, independently of any binding partners [21], and interact with most known +TIPs, thus directing them to MT plus ends (reviewed in [19]). Protein depletion and rescue experiments showed that EB1 and EB3, but not EB2, promote persistent MT growth by suppressing catastrophes [12]. EBs track growing plus ends via their N- terminal MT-binding domain [12], which is highly conserved and adopts a ‘calponin homology’ (CH) fold [22]. The C-terminus of EBs interact with a large range of structurally diverse +TIPs, and is composed of an alpha-helical coiled-coil domain that mediates EB dimer formation [23], [24], and an acidic C-terminal tail of low complexity that includes a C-terminal EEY/F motif, like that found in alpha-tubulin [25].

Interactions between EB1 and its partners can be broadly classified into two groups based on their modes of interaction. First, the cytoskeleton-associated protein glycine-rich (CAP-Gly) domains of CLIP-170 and the large subunit of the dynactin complex p150glued specifically recognize the EEY/F motif of EBs [26]. The second mechanism of EB-partner interaction is mediated via a unique EBH domain of EBs (Conserved domain Database pfam03271), which partly overlaps with the coiled-coil domain. A large family of +TIPs (reviewed in [19], [27]), interacts with the EBH domain of EBs via a conserved S-x-I-P motif (SxIP, where x is any amino acid) that is embedded within a region of low complexity that is rich in basic, serine and proline residues. The complexity of the +TIP interactome was recently confirmed in a proteome-wide screen for mammalian SxIP motif-containing +TIPs, in which biochemical and bioinformatics approaches were combined [28].

In the present work, we report that p104, a protein previously described as a *Theileria* sporozoite microneme-rhoptry protein, is a major schizont surface protein that functions as a bona fide EB1-binding protein, interacting with EB1 via a classic ‘SxIP’ motif. In this capacity, p104 is the first parasite-encoded EB1-binding protein described to date.

## Results

### P104 is a major surface protein of *T. annulata* schizonts

**Figure 1:**
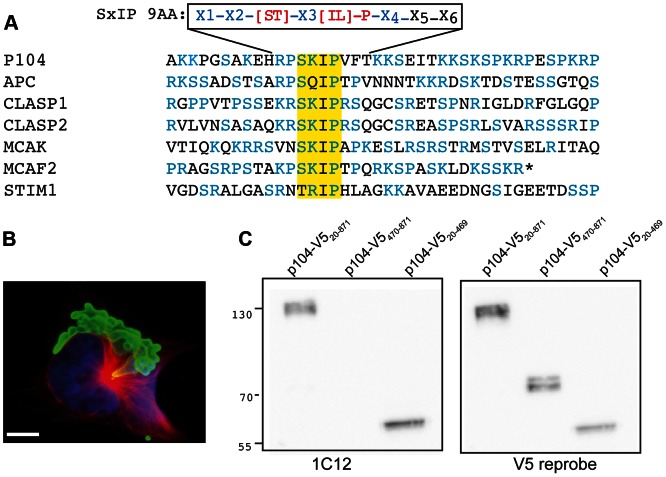
The *T.* *annulata* schizont surface protein p104 contains an SxIP motif. (A) Alignment of p104_554–593_ with the corresponding sequences from human APC, CLASP1, CLASP2, MCAK, MCAF2 and STIM1. The SKIP motif is highlighted in yellow, serines, prolines and basic residues (K/R) in blue. The recently expanded nine-amino acid SxIP motif (SxIP 9AA) defined by Jiang et al. [28] is included for reference. (B) Image showing how mAb 1C12 (green) labels the schizont surface; MTs are stained with anti-tubulin (red). DNA is stained with DAPI (blue). Scale bar = 5 µm. (C) Immunoblot analysis of lysates prepared from COS-7 cells expressing V5-tagged p104 fragments (full-length; p104_20–871_-V5, C-terminal; p104_470–871_-V5, or N-terminal; p104_20–469_-V5). Blots were probed with 1C12 and reprobed with anti-V5.

A bioinformatics search was performed to identify schizont surface proteins that have the potential to interact with EBs. As the presence of a GPI-anchor is a reliable parameter for surface expression, *T. annulata* GeneDB (old version) was queried for genes encoding proteins containing a predicted signal peptide and GPI-anchor sequence (for detailed information, see Table S2 and Figure S5, upper section). This resulted in 19 candidates that were screened for the presence of an SxIP motif and representation in a *T. annulata* schizont proteome database obtained by mass spectrometry [29]. We identified *TA08425*, annotated in *Theileria annulata* GeneDB as encoding ‘*Theileria parva* microneme-rhoptry antigen of 104 kDa’ (henceforth referred to as ‘p104’), as the only candidate. The protein encoded by *TA08425* (accession number XM_948006) shows 51% identity and 65% similarity to *T. parva* p104 and possesses a predicted signal peptide and putative GPI anchor signal. Even though p104 was originally identified as a protein expressed by *T. parva* sporozoites [30], *T. annulata* p104 could also detected by mass spectrometry in Triton X-114 lysates of purified *T. annulata* schizonts enriched for membrane proteins [29]. Sequence analysis of *p104* (as isolated from TaC12 cells; Figure S1) predicted a protein with a globular N-terminal domain and a highly disordered C-terminal region of low sequence complexity that is rich in basic, serine and proline residues. The N-terminal half of the protein contained four so-called ‘FAINT (Frequently Associated in *T* *heileria*) domains’ (InterPro domain DUF529, IPR007480), a highly polymorphic domain with an average length of 70 residues [31]. FAINT domains are expanded in a lineage-specific manner in *Theileria* and characteristic of proteins that represent the schizont secretome [32]. Embedded within the basic-S/P rich C-terminal region, we identified an ‘SxIP’ motif, characteristic of EB1-binding proteins [33]. The p104 SxIP motif also fulfilled the contextual conditions outlined by Jiang et al, which stipulates that in the sequence X1-X2-[ST]-X3-[IL]-P-X4-X5-X6, at least one of the residues X1 to X4 should be an R or K and none of the residues X1 to X6 should be a D or E [28]. An alignment of the corresponding domains for a number of EB1-binding proteins is shown in Figure 1A.

A recent screen of a SMART cDNA library derived form *T. annulata* (Ankara) schizont cDNA [31] (kind gift of Dr. Gordon Langsley) revealed that the mAb 1C12 recognizes p104. 1C12 is one of a panel of mAbs obtained after immunization of mice with *T. annulata* (Hissar)-infected leukocytes [34]. This mAb recognizes *T. annulata* schizonts, but not merozoites and piroplasms [34], [35], and serves as a useful diagnostic marker for the parasite [5], [6], [36]. The identity of this immunodominant surface antigen, however, was unknown until now. Immunofluorescence microscopy (IFM) using the mAb 1C12 showed that p104 is expressed abundantly on the surface of the schizont (Figure 1B).

*p104* cloned by PCR from genomic DNA isolated from the *T. annulata*-transformed cell line TaC12 differed from the *T. annulata* Ankara sequencing strain [31]. A comparison of the predicted proteins (AGB56140.1 and XP_953099.1) yielded 92% identities. We performed all of our experiments using TaC12 cells, and all of the expression constructs used in this study were based on the TaC12 *p104* sequence (Figure S1, submitted to GenBank JX965955). In order to ensure cytoplasmic expression in mammalian cells, the sequence encoding the predicted signal peptide or GPI anchor sequences were omitted in expression vectors. Western blot analysis of lysates of COS-7 cells expressing V5-tagged ‘full-length’ (amino acids 20-871), C-terminal (p104-CT, 470-871) and N-terminal (p104-NT, 20-469) p104 fragments revealed that 1C12 recognizes an epitope in the N-terminal portion of p104 (Figure1C).

### Localization of *T. annulata* p104 to MT plus ends depends on an SxIP motif

**Figure 2:**
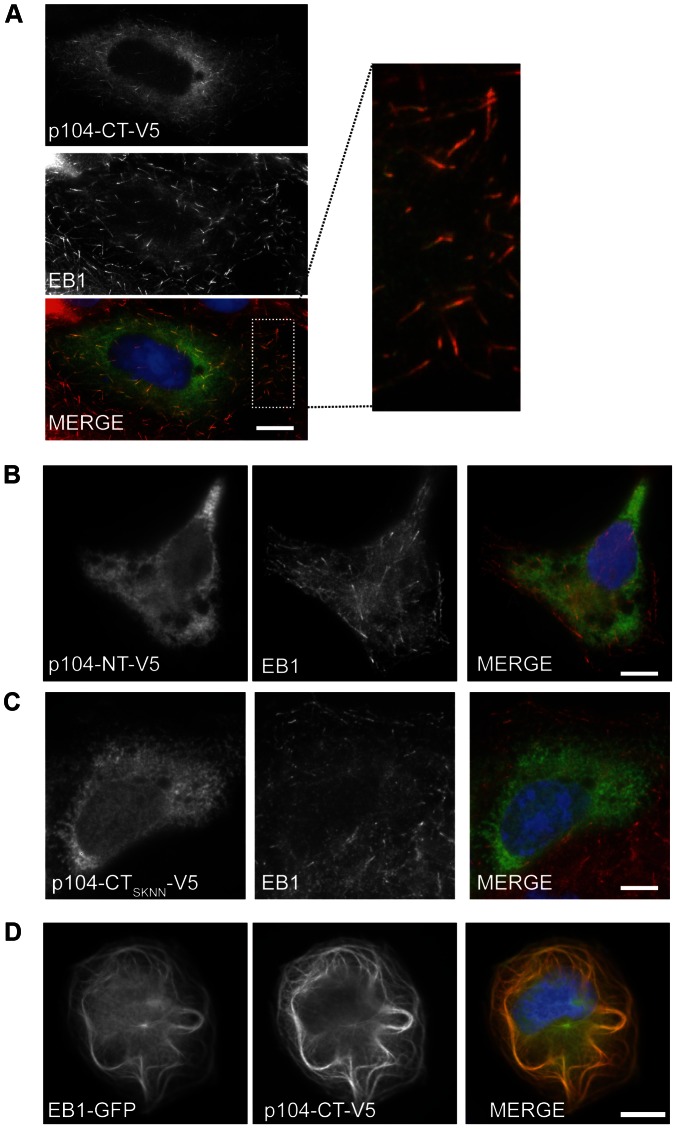
p104-V5 co-localization with endogenous EB1 at MT plus ends requires an intact SxIP motif. (A) Image of a COS-7 cell transiently expressing p104-CT-V5, fixed with ice-cold methanol and stained with anti-EB1 (red) and anti-V5 (green); the white rectangle indicates the magnified cytoplasmic region. DNA is stained with DAPI (blue). Scale bar = 10 µm. (B) The N-terminal region of p104 (p104-NT-V5) does not localize to MT plus ends. (C) Mutation of the SKIP motif to SKNN abolishes plus end localization. (D) Image of over-expressed p104-CT-V5 co-localizing with EB1-GFP at bundled MTs in COS-7 cells.

To investigate if *T. annulata* p104 has the potential to interact with cytoskeletal structures of the mammalian cell, we transiently expressed soluble V5-tagged p104 fragments corresponding to residues 20-469 (p104-NT-V5) and residues 470–871, (p104-CT-V5) in COS-7 cells, and analyzed their localization by IFM. V5-tagged p104-CT co-localized clearly with endogenous EB1 at MT plus ends (Figure 2A), displaying the typical ‘comet-like’ dashes, characteristic of EB1 and other +TIPs [37]. This pattern of expression was also detected when the full-length p104 (20 – 871) was expressed (not shown). Expression of p104-NT-V5, on the other hand, resulted in a diffuse cytosolic localization (Figure 2B). Importantly, MT plus end localization of p104-CT-V5 was abolished upon mutation of the SKIP motif to SKNN (p104-CT_SKNN_) (Figure 2C), indicating that p104 association with MT plus ends depends on a functional SxIP-motif. Overexpression of EB1 is known to result in MT bundling [38]. Co-expression of p104-CT-V5 with EB1-GFP resulted in the co-localization of both proteins to bundled MTs (Figure 2D). A detailed genome-wide bioinformatics screen of the *T. annulata* predicted proteome revealed the presence of more than 200 proteins containing an SxIP motif (see lower section of Figure S5, Table S3 and discussion below). Only a minority of these possesses a predicted signal peptide. Three of these candidates were expressed as V5-tagged proteins in COS-7 cells. TA20980 contains two SxIP motifs; TA17545 is a member of the subtelomere-encoded variable secreted protein (SVSP) family [31] and TA17375 is known as p150 (polymorphic antigen precursor in *T. parva* [39]. Interestingly, in contrast to p104-CT-V5, none of the proteins colocalised with EB1 or showed the typical comet-like dashes, despite possessing credible SxIP motifs (Figure S2)

### EB1 association with the *T. annulata* schizont surface is independent of MTs and requires the C-terminal ‘End-binding homology’ domain

**Figure 3:**
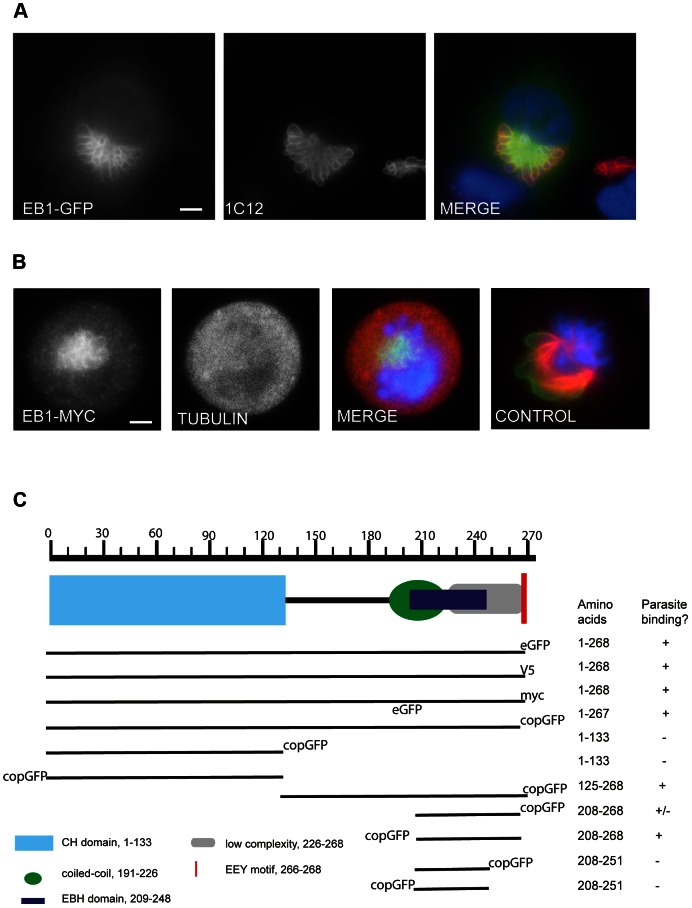
EB1 interacts with the schizont surface in a MT independent manner, via its End-binding homology (EBH) domain. (A) Image of TaC12 cell expressing EB1-GFP; the schizont was stained using 1C12 (red). DNA is stained with DAPI (blue). Scale bar = 5 µm. (B) EB1 binding to the schizont does not require MTs: TaC12 cells expressing EB1-myc were treated with 0.1 µg/ml nocodazole for 16 h, collected by mitotic shake-off, fixed with 4% PFA on ice, and stained with anti-myc (green) and anti-tubulin (red) antibodies. The last panel (CONTROL) shows a cell in prometaphase that was not treated with nocodazole, to point out the effect of nocodazole on depolymerizing MTs. DNA is stained with DAPI (blue). Scale bar = 5 µm. (C) Truncation analysis of EB1 binding to the schizont surface. TaC12 cells were transfected with plasmids encoding either full-length GFP-, V5- or myc-tagged EB1, or deletion constructs encoding different domains of EB1 tagged with copGFP.

To determine whether EB1 can interact with the parasite surface, we transiently expressed GFP-tagged EB1 and EB3 (a kind gift from Anna Akhmanova) in TaC12 cells. EB1-GFP labeled the entire surface of the schizont (Figure 3A). The same pattern could be observed using plasmids encoding EB1 containing a C-terminal myc or V5 tag (not shown) confirming that the observed localization of ectopically expressed EB1 is not an artifact caused by the GFP tag. In live imaging experiments, EB1-GFP-labeled ‘comets’ were readily detected moving from the centrosome to the periphery as reported [37], along with a striking association of EB1-GFP with the parasite surface (Movie S1), ruling out the possibility that the association of over-expressed EB1 with the schizont was a fixation artifact. Transfection experiments using EB3-GFP revealed that EB3, which shows overlapping functions with EB1 [12], [25], [40], also associates with the schizont surface (Figure S3).

To verify whether EB1 recruitment to the schizont requires MTs, TaC12 cells transiently expressing EB1-myc were treated with nocodazole, a drug that inhibits MT polymerization. Cells exposed to the drug (0.1 µg/ml) for 16 h completely lacked MTs and were arrested in prometaphase (an image of a prometaphase cell that was not subjected to nocodazole treatment and possesses intact MTs is included for comparison). Under these conditions, EB1-myc could still be found in association with the schizont, suggesting that the interaction of EB1 with the schizont is independent of MTs (Figure 3B). To explore the mode of EB1 interaction with the parasite surface, we generated EB1 truncation mutants and monitored the association of transiently expressed GFP-tagged EB1 fragments with the schizont surface by live imaging and IFM (Figure 3C). The C-terminal tyrosine residue of EB1 was found to be dispensable for parasite-localization, suggesting that binding to the schizont did not involve a ligand containing a CAP-Gly domain. The C-terminal portion of EB1, containing the linker region, coiled-coil dimerization domain, and the EBH domain (EB1_125–268_–GFP), was sufficient to bind to the parasite surface, while the N-terminal MT binding domain (EB1_1–133_–GFP) did not localize to the schizont. These data suggested that the EB1-schizont interaction is dependent on the EBH domain of EB1, and likely is mediated via an SxIP-containing protein. An intact dimerization domain of EB1 was also dispensable for schizont interaction, as EB1_208–268_, a fragment consisting of the EBH domain and the acidic C-terminal tail, was sufficient to bind to the schizont. The position of the copGFP tag influenced the extent to which EB1_208–268_ associated with the schizont. When EB1_208–268_ was fused to copGFP at its N-terminus, all transfected cells showed clear parasite labeling; when the tag was placed at the C-terminus, however, staining patterns were more variable, with only few transfected cells showing labeled schizonts. Finally, we determined that EB1_208–251_, defined as the shortest EB1 fragment capable of interacting with APC [41], failed to associate with the schizont.

### The SKIP motif of p104 is required for interaction with endogenous EB1

**Figure 4:**
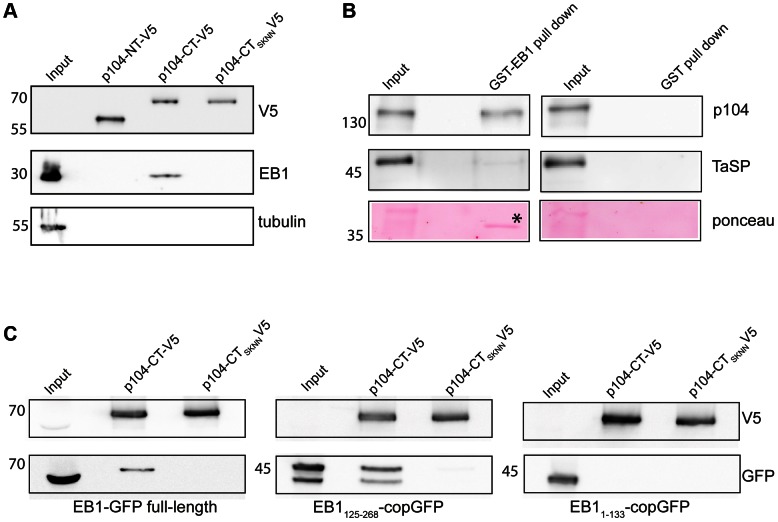
The SKIP motif is required for p104 interaction with EB1. (A) COS-7 cell lysates were incubated with Halo-tagged p104-CT-V5, p104-NT-V5 and p104-CT_SKNN_-V5 linked to resin. The resin was washed with a high salt wash buffer and proteins eluted from the Halolink resin by cleavage with TEV protease. Eluates were subjected to immunoblot analysis using anti-V5 (to reveal p104-NT-V5, p104-CT-V5 and p104-CT_SKNN_-V5 in the eluates), anti-EB1 (to monitor EB1/p104 interaction) and anti-tubulin (as a control). (B). Lysate from TaC12 cells was subjected to pull-down using GST-EB1 or GST alone as a negative control. EB1 and its potential binding partner(s) was cleaved off GST-EB1 using precision protease and subjected to SDS-PAGE (10% gel) followed by immunoblot analysis using anti-p104 (1C12, upper panels) and anti-TaSP (middle panels). Control samples (right) were treated in the same manner. Ponceau staining (lower panels) shows cleaved-off EB1 (marked by asterisk). (C) Lysates of COS-7 cells expressing full-length EB1-GFP, EB1_125–268_-GFP and EB1_1–133_-GFP were subjected to pull-down analysis using recombinant p104-CT-V5 and p104-CT_SKNN_-V5 as described above. EB1-GFP and EB1_125–268_-GFP interacted with p104-CT-V5. EB1_1–133_-GFP failed to interact with p104-CT-V5, and p104-CT_SKNN_-V5 did not interact with any EB1 fragments. The lower panels represent immunoblots probed with anti-GFP to demonstrate the presence of EB1-GFP fragments in pull-down elutions. The top panel shows the same blot reprobed with anti-V5 to demonstrate the presence of bait proteins. EB1_125–268_-GFP is found as a doublet in transfected COS-7 cells, likely due to the presence of a second in-frame translational start codon. In all panels, 1% of the lysate (10 µl of 1 ml) was loaded as input; pull-down lanes represent 10% of eluted protein.

We next carried out pull-down experiments to confirm that *T. annulata* p104 is a functional EB1 binding partner. Recombinant Halo-tagged p104 fragments containing a C-terminal V5 tag were produced in *E. coli* and used for pull-down analysis of lysates prepared from COS-7 cells. Recombinant p104-CT migrates more slowly in SDS-PAGE than predicted (70 kDa rather than the predicted 46 kDa), while the N-terminal fragment has an apparent molecular weight as expected of 55 kDa (Figure 4A and 1C). Endogenous EB1 was detected after pull-down with recombinant p104-CT, but not with p104-NT. Mutation of the SKIP domain to SKNN (p104-CT_SKNN_) abolished the interaction between EB1 and p104-CT, supporting our observations made by IFM that p104 can interact with EB1, and that this interaction is mediated via the SKIP motif. Conversely, in pull-down assays performed on lysates of *T. annulata*-infected TaC12 cells, GST-tagged EB1 produced in *E. coli* bound parasite-derived p104 (Figure 4B), but not TaSP, another major schizont surface protein [7].

Further biochemical evidence for a specific p104/EB1 interaction was obtained by subjecting lysates of COS-7 expressing EB1-GFP fragments to pull-down experiments with recombinant p104-CT (Figure 4C). While full-length EB1-GFP and EB1_1–133_-GFP migrated at the predicted molecular weight of 60 kDa and 45 kDa respectively, EB1_125–268_-GFP, predicted to be 46 kDa, was resolved as two bands of approximately 40 and 50 kDa, likely due to the presence of a second in-frame translational start codon. In agreement with our observations made by IFM, full-length EB1-GFP and EB1_125–268_-GFP, but not EB1_1–133_-GFP, bound to p104-CT. No binding occurred to p104 containing a mutated SKIP domain (p104-CT_SKNN_).

### p104 shows MT plus end tracking in COS-7 cells

To identify the region of p104 required for EB1 interaction in more detail, we expressed a fragment of p104, (p104_521–634_) consisting of 114 aa and containing the SKIP motif, in mammalian cells. In transfected COS-7 cells, GFP-p104_521–634_ tracked MT plus ends, producing ‘comet-like structures’ characteristic of EB1 and many EB1-binding proteins (Movie S2) [12], [33], [42]. The plus end tracking behavior of GFP-p104_521–634_ was completely abolished upon mutation of the SKIP motif to SKNN (not shown). To further define the region of p104 required for plus end tracking, we generated recombinant GFP-tagged p104 fragments of 40 aa (GFP-p104_554–593_) for expression in mammalian cells, and also in *E. coli* for microinjection experiments. The p104_554–593_ aa sequence corresponds to that shown in the alignment in Figure 1A. GFP-p104_554–593_ tracked growing MT plus ends in transfected COS-7 cells (not shown), and strongly labeled the centrosome of microinjected TaC12 cells (Movie S3). GFP-p104_ 554–593_ tracked growing MTs emanating from the centrosome (Movie S3) and also tracked MT plus ends along the surface of the schizont, in the same manner as EB1-GFP (compare with Movie S1). In mitotic cells, GFP-p104_554–593_ localized to centrosomes and to the tips of growing spindle MTs (not shown), as was reported for EB1-GFP [43].

### Host cell EB1 associates with the *Theileria* schizont in a cell cycle dependent manner

**Figure 5:**
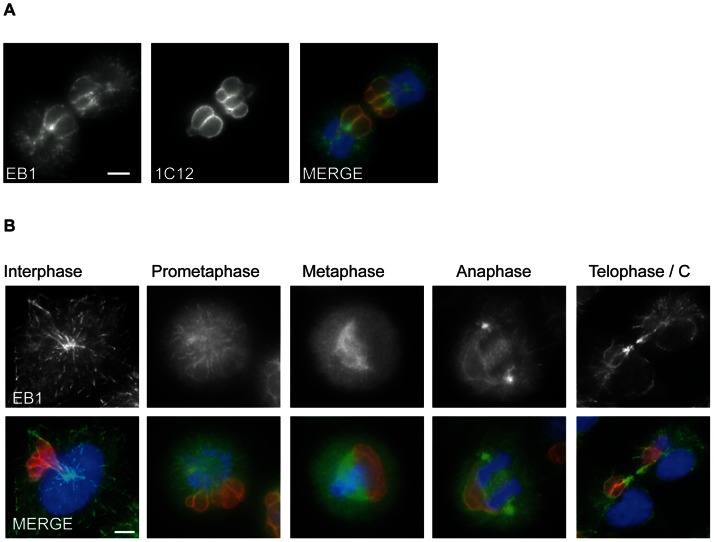
Host cell EB1 interaction with the schizont surface. (A) TaC12 cells were fixed with ice-cold methanol, and sequentially stained with rat mAb anti-EB1 (green) and anti-p104 (1C12; red). DNA is stained with DAPI (blue). (B) TaC12 cells were synchronized in specific cell cycle stages and stained with anti-EB1 (green) and anti-p104 (1C12, red) as above. Scale bars = 5 µm.

Having shown that p104 and EB1 interact in an SxIP-dependent manner, and that ectopically expressed EB1-GFP associates with the schizont, we focused our attention on endogenous host cell EB1 in *T. annulata*-transformed cells. In IFM preparations fixed with methanol, EB1 was detectable as ‘comet-like’ fluorescent dashes at the plus ends of MTs and at the centrosome, as described in other cell-lines [37] [44]. While the extent to which EB1 association with the schizont varied within a mixed population of cells, we noticed a striking association of EB1 with the schizont in cells undergoing cell division (Figure 5A, and 5B last panel). In cells in metaphase, however, EB1 was largely undetectable at the parasite surface.

Because mitotic cells were detected only infrequently in an unsynchronized culture, we made use of various cell synchronization protocols to analyze in more detail the association of EB1 with the schizont. Cells were first synchronized in prometaphase by overnight nocodazole treatment and then released from nocodazole block into media containing the proteasomal inhibitor MG132 that blocks the degradation of cyclin B, thus synchronizing cells in a metaphase-like state. Images representative for the different cell cycle stages are presented in Figure 5B. In interphase cells, which have a more flattened morphology, the characteristic ‘comet-like’ distribution of EB1 was clearly noticeable and, in many cells, the outline of the schizont was labeled with EB1 (Figure 5B, first panel). EB1 also decorated the surface of the schizont during prometaphase. As cells progressed through metaphase, however, schizont staining was notably absent whereas the mitotic spindle was strongly labeled. Upon release from metaphase arrest, EB1 re-associated with the schizont surface and parasite-associated EB1 could easily be detected in cells in anaphase and telophase. EBs are very conserved and the *T. annulata* genome contains a gene (*TA18025*) encoding EB1, which shows up to 49% positivity when compared with bovine EB1. Recombinant TaEB1 subjected to immunoblot analysis failed to react with the anti-EB1 antibody (KT51), and no reactivity could be demonstrated with lysates of purified parasites, thus excluding cross-reactivity (Figure S4).

### p104 is phosphorylated in a cell cycle-dependent manner

**Figure 6:**
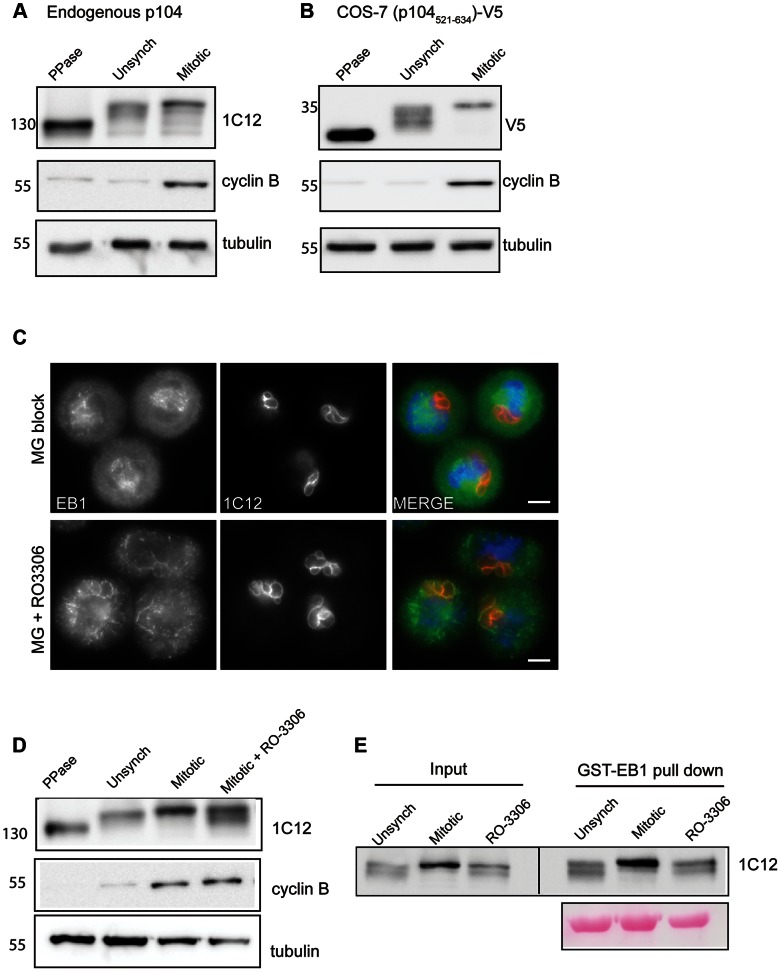
p104 phosphorylation changes in a cell cycle-dependent manner. (A) Immunoblot showing increased p104 phosphorylation during mitosis. 6% SDS-PAGE gels were utilized, allowing multiple forms of p104 phosphorylation to be detected. Mitotic TaC12 cells were obtained by mitotic shake-off and subsequent incubation for 1 h with the proteasomal inhibitor MG132. Lysates were subjected to immunoblot analysis using anti-p104 (1C12). ‘PPase’ indicates that lysate was treated with lambda phosphatase. Increased levels of cyclin B reflect synchronization in mitosis; tubulin was used as a loading control. (B) During mitosis, significant phosphorylation occurs in the vicinity of the SKIP motif. COS-7 cells expressing p104_521–634_-V5 were synchronized in mitosis and lysates analyzed as above. (C) Localization of EB1 at the schizont surface correlates inversely with Cdk1 activity. TaC12 cells were synchronized in a metaphase-like state by mitotic shake-off followed by treatment with the proteasomal inhibitor MG132. Cdk1 inhibition (RO-3306, 10 µM) was carried out for 30 min in the presence of MG132 prior to fixation with ice-cold methanol. Cells were stained sequentially with rat anti-EB1 (green) and anti-p104 (1C12, red). DNA is stained with DAPI (blue). Scale bar = 5 µm. (D). Immunoblot showing altered phosphorylation following Cdk1 inhibition. Mitotic TaC12 cells were obtained as described above. Cdk1 activity was inhibited in mitotic cells by the addition of RO-3306 (10 µM) for 30 minutes (mitotic + RO-3306). Lysates were subjected to immunoblot analysis using anti-p104 (1C12), anti-cyclin B and anti-tubulin. (E). Pull-down analysis reveals that both phosphorylated and unphosphorylated p104 can interact with recombinant EB1. Lysates were prepared from unsynchronized, mitotic and Cdk1-inhibited TaC12 cells. as described, and subjected to pull-down using GST-EB1. EB1 and its potential binding partner(s) was cleaved off GST-EB1 using precision protease and subjected to SDS-PAGE (6% gel) followed by immunoblot analysis using anti-p104. Ponceau staining (lower panel) shows cleaved-off EB1. 1% of the lysate (10 µl of 1 ml) was loaded as input; pull-down lanes represent 10% of eluted protein.

Endogenous *T. annulata* p104 has a predicted molecular weight of 101 kDa, but migrates in SDS-PAGE with an apparent molecular mass of approximately 130 kDa. As p104 contains multiple predicted phosphorylation sites (NetPhos 2.0), we investigated whether p104 is phosphorylated in TaC12 cells. Incubation of lysates from unsynchronized TaC12 cells with λ-phosphatase caused a significant downshift in the apparent molecular weight of p104, indicating that endogenous p104 is extensively phosphorylated (Figure 6A). While p104 appears to be highly phosphorylated throughout the duration of the cell cycle, we noticed a slight up-shift in endogenous p104 migration in lysates from mitotic cells (those arrested in metaphase), compared to lysates prepared from unsynchronized cells (Figure 6A). To obtain more detailed information on p104 phosphorylation in the region important for EB1 interaction, we subjected lysates from cells expressing p104_521–634_-V5 to λ-phosphatase treatment and subsequent western blot analysis. A significant downshift was observed upon phosphatase treatment, demonstrating that phosphorylation occurs within this region. Furthermore, the difference in protein migration pattern of p104_521–634_-V5 observed between unsynchronized and mitotic cells was more striking than for endogenous p104, suggesting that extensive cell-cycle dependent phosphorylation occurs in this region (Figure 6B). Within this 114 amino acid stretch, there are 6 ‘strong’ Cdk1 phosphorylation sites (S/T*-P-x-K/R), and three additional ‘weak’ Cdk1 phosphorylation sites (S/T*-P) [45]. Preliminary LC-MS/MS analysis revealed serine phosphorylation of at least 4 of these sites (serines 589, 601, 607 and 622). Treatment of TaC12 cells synchronized in mitosis with the Cdk1 inhibitor RO-3306 resulted in a partial, but pronounced, reduction in p104 phosphorylation (Fig. 6D). The state of p104 phosphorylation does not appear to affect its interaction with recombinant EB1, however, as, p104 present in lysates prepared from mitotic as well as unsynchronized or RO-3306-treated cells was capable of binding to GST-EB1 in pull-down experiments (Fig. 6E).

The association of EB1 with the schizont appeared to correlate inversely with the pattern of Cdk1 activity, with EB1 being undetectable at the schizont surface during metaphase, when Cdk1 activity peaks. To investigate this further, we analyzed the effect of Cdk1 inhibition on the localization of EB1 with the schizont. As mentioned before, in cells synchronized in metaphase, EB1 was found to localize to the mitotic spindle, but could not be detected at the schizont surface. Blocking Cdk1 by treatment with the inhibitor RO-3306 resulted in the induction of furrow ingression, and a pronounced recruitment of EB1 to the schizont surface. This indicates that EB1 interaction with the schizont is, at least to some degree, negatively regulated by Cdk1 (Figure 6C).

### p104 targeted to mitochondria induces EB1 mislocalization

**Figure 7:**
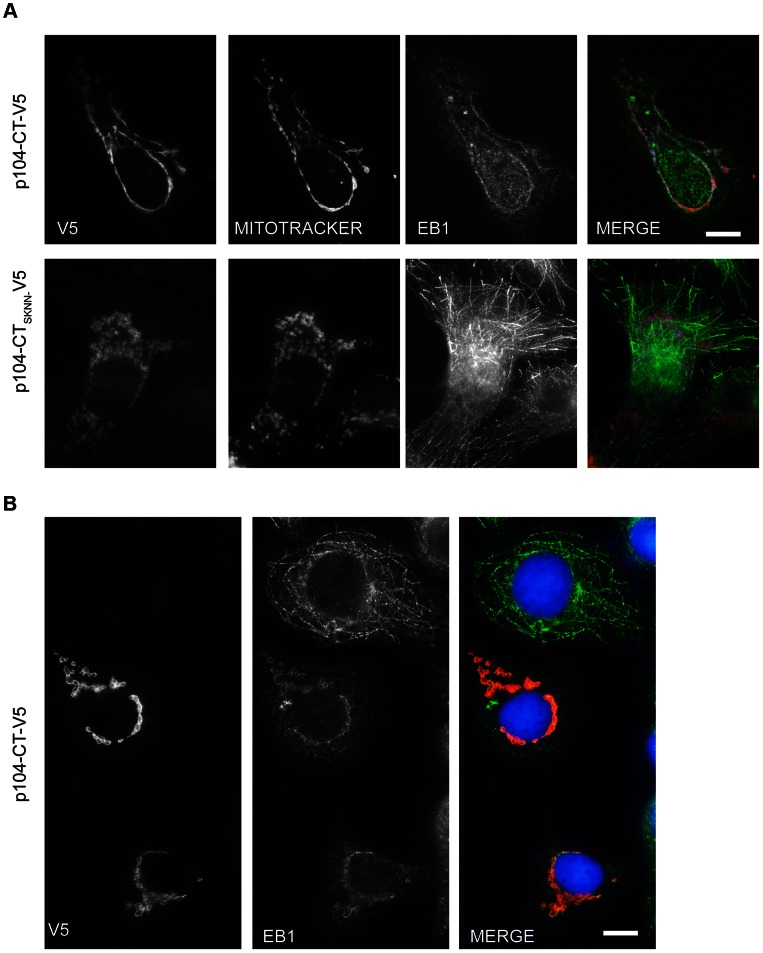
p104 targeted to mitochondria results in EB1 mislocalization. (A) Images of COS-7 cells expressing either p104-CT-V5 (top panel) or p104-CT_SKNN_-V5 (lower panel) fused to the mitochondrial targeting sequence of vaccinia virus protein F1L. Mitochondria were visualized using Mitotracker (red) 24 h post transfection prior to fixation in ice-cold methanol and staining with rat anti-EB1 (green) and mouse anti-V5 (white). (B) Image showing 2 transfected cells expressing p104-CT-V5 targeted to mitochondria and one untransfected cell (top); transfected cells were fixed with ice-cold methanol and stained with rat anti-EB1 (green) and mouse anti-V5 (red). DNA is stained with DAPI (blue); Scale bar = 10 µm. Note the presence of MT-associated EB1 in the untransfected cell.

Next we aimed to establish whether membrane-anchored p104 is capable of recruiting endogenous EB1 independently of other parasite proteins. To that extent, p104-CT was fused to the the C-terminal tail of the vaccinia virus F1L protein which targets the outer membrane of mitochondria [46]. V5-tagged p104-CT was targeted to COS-7 cell mitochondria and EB1 localization monitored by IFM. In transfected cells in which p104-CT was clearly visible at the mitochondria, EB1 was also notably re-distributed to the mitochondria (Figure 7A) Interestingly, in most instances, EB1 mislocalization was accompanied by reduced MT plus end binding. Importantly, whereas p104-CT-SKNN was also targeted to the mitochondria, it failed to recruit EB1 and, in such cells, EB1 localized correctly to MTs (Figure 7A lower panel). Figure 7B shows an area containing two transfected cells and one untransfected cell for comparision, demonstrating that in untransfected cells, EB1 was normally distributed to the plus ends of MTs. These data show that membrane-bound p104 functions as an EB1-recruiting protein, independently of other parasite proteins.

## Discussion

In a process known as dynamic instability, MTs alternate between phases of growth and shrinkage, allowing them to ‘explore’ different regions of the cytoplasm, and contribute to intracellular organization, cellular function and polarity. The *Theileria* schizont taps into this network, and the interaction between the schizont and host cell MTs during mitosis and cytokinesis is crucial for the persistence of the parasite in its transformed host [5]. Considering the intricate interactions observed during mitosis and cytokinesis [5], the association between the schizont and host cell microtubular network can be expected to be complex involving different host and parasite factors. The +TIP interaction network is large and dynamic, and EB1 fulfills the role of ‘master regulator’. Using pull-down approaches as well as co-localization and MT plus end tracking assays we now demonstrate that p104, a major *T. annulata* schizont surface protein, is a genuine EB1-binding protein of the SxIP family. We posit that, by providing docking sites for EB1, p104 contributes to capturing growing MTs emanating from the centrosomes in interphase cells and from spindle poles during mitosis. Whereas our bioinformatics search (using http://old.genedb.org/genedb/annulata) did not predict the presence of other GPI-anchored schizont proteins containing a potential SxIP motif, our findings do not exclude the possibility that other parasite surface proteins, or proteins secreted by the schizont, participate in EB1 binding. SxIP motifs can be found in a wide range of proteins in different organisms including *Theileria*. For instance, a bioinformatics analysis of two transforming (*T. annulata* and *T. parva*) and one non-transforming *Theileria* (*T. orientalis*) yielded 217, 201 and 154 proteins with predicted SxIP motif, a number of which also contained a predicted signal peptide (see Figure S5 and Table S3). At this stage, it is not clear if the reduced number of SxIP motif-containing proteins in *T. orientalis* reflects different requirements for EB binding partners in the parasite's life cycle. Whether any of these many candidate proteins function as genuine EB1-binding partners that could potentially interact with host cell EB1 can only be established experimentally. This is underpinned by our observation that TA20980, TA17545 and TA17375 all failed to co-localize with EB1 at MT plus ends in COS-7 cells, despite the presence of convincing SxIP-motifs.

While EB1 might facilitate the initial interaction of MTs plus ends with the schizont, it can be assumed that additional molecules of parasite and/or host cell origin are required to stabilize MTs at the schizont surface. Preliminary observations suggest that host cell CLASP1, a protein with known MT-stabilizing activity [14], could contribute to MT stabilization at the parasite surface. In this context, we recently found CLASP1 to be constitutively recruited in abundant amounts to the schizont surface (unpublished). How CLASP1 associates with the parasite surface is presently under investigation. To what extent TaSP [7] or gp34 [6] are potentially involved in these processes is presently unclear.

The identification of p104 as a major schizont surface protein was surprising. In earlier work, p104 was described as a strongly antigenic protein localized in the microneme-rhoptry complexes of sporozoites [30]. Mass spectrometric analysis [29] and screening of a cDNA expression library prepared from schizonts, however, confirmed that p104 is also expressed by the transforming schizont. We also established that the mAb 1C12, used as a useful diagnostic tool and marker of the parasite [36], recognizes the N-terminal region of p104.

When expressed as a soluble protein in COS-7 cells, p104 shows MT plus end tracking, typical of +TIPs. The fact that the p104 EB1 binding domain encompassing the SKIP motif could be reduced to as little as 40 aa excludes a role in EB1 recruitment of the FAINT domains, contained in the N-terminal half of p104. Importantly, in all experiments, p104/EB1 interaction was critically dependent on an intact SKIP motif and mutation to SKNN abrogated EB1 binding in all experiments.

For a number of +TIPs, the affinity for EB1or MTs is regulated by phosphorylation in a spatio-temporal manner [47]. For example, MCAK binding to EB1 is negatively regulated by Aurora B phosphorylation [33], and Cdk1-dependent phosphorylation of SLAIN2, a protein that links MT plus end–tracking proteins and controls MT growth during interphase, disrupts its binding to EB1 during mitosis [42]. In the case of CLASP2, multisite Cdk- and GSK3-dependent phosphorylation was found to fine-tune the strength of CLASP2/EB1 interaction [48], [49]. Like many +TIPs, p104 is also phosphorylated. Phosphorylation is extensive and cell cycle-dependent changes could be observed. Upon treatment with the Cdk1 inhibitor RO-3306, pronounced alterations in the p104 phosphorylation pattern were observed. Phosphorylation was not completely abrogated, however, indicating that p104 is also a substrate for kinases other than Cdk1. The exact role of p104 phosphorylation in EB1/p104 interaction is still unresolved. Pull-down experiments revealed that different phosphorylated forms of p104 can bind GST-EB1. In this regard, p104 differs markedly from SLAIN2, which, in its hyperphosphorylated state, fails to bind to GST-EB1 [42].

While inhibition of the mitotic kinase Cdk1 clearly resulted in increased EB1 accumulation at the schizont surface, our observations indicate that the cell cycle-dependent interaction of EB1 with the schizont surface during mitosis is regulated in a more complex manner than merely through a selective, phosphorylation-dependent interaction of EB1 with p104.

Endogenous EB1 may differ substantially from recombinant EB1 used in pull-down experiments and display altered ligand-binding properties during the cell cycle. The spatiotemporal regulation by which EB1 is recruited to different host cell structures involves its interaction with specific proteins that are often modified in a cell cycle-dependent manner [47]. It is worth noting, however, that EB1 itself also undergoes cell cycle-specific modifications. This includes acetylation of K220 in the EBH domain, which has been shown to prevent EB1 interaction with various SxIP proteins [50]. Significantly, Xia and colleagues found that K220 acetylation is readily apparent in metaphase cells and decreased in anaphase cells. EB1 is not detected at the parasite surface during metaphase, but reappears at anaphase. Considering the striking coincidence, it is conceivable that EB1 interaction with the parasite is also regulated by acetylation. The dynamic acetylation of EB1 was found to help orchestrate accurate kinetochore/MT interactions and acetylation [50]. Thus, while it is required to contribute to important mitotic functions – such as the alignment of host cell chromosomes in preparation for exit from mitosis [43], [51] - EB1 might be transiently inaccessible for the parasite.

That EB1 dissociates from the parasite surface during metaphase could be considered counterintuitive. The transient lack of EB1 association with the schizont surface is not accompanied by a loss of MTs, however. On the contrary, we have shown that the parasite remains tightly associated with host cell MTs throughout mitosis [5] (see also the control panel in Fig. 3B). During anaphase, EB1 might contribute to the docking of central spindle MTs to the parasite surface, which we have shown to be essential for the distribution of the parasite over the two daughter cells [5].

Cell cycle-dependent recruitment of host cell proteins to the schizont surface is not without precedent. A similar phenomenon has been observed for host cell Plk1, another protein important for mitotic progression. Plk1 associates with the parasite surface during G2 and early mitosis, is released from the parasite surface during prometaphase, and promptly re-associates as the cell enters anaphase [5].

Although our observations strongly indicate that p104 acts as an EB1-binding protein, up till now, we have failed to interfere with EB1 binding to the schizont surface. Following transfection of TaC12 cells with mitochondria-targeted p104, very few (<0.1% of those transfected) surviving cells were recovered rendering a meaningful analysis impossible. Overexpression by transient transfection or microinjection of soluble p104 (GFP-p104_554–593_) failed to disrupt EB1/schizont interaction. GFP-p104_554–593_ plus end tracking in microinjected *T. annulata-*infected cells resembled that observed for EB1-GFP, and GFP-p104_554–593_-labeled MT plus ends could also be observed tracking along the parasite surface, suggesting that these short p104 fragments had neither a dominant negative effect on the association of EB1 with MT plus ends nor on EB1 binding to the parasite surface. One explanation could be that membrane-bound p104 binds EB1 with higher affinity than soluble p104. That membrane-bound p104 can indeed provide a strong docking site for EB1 is reflected by the fact that EB1 was extensively mislocalized in cells expressing p104 targeted to the outer membrane of mitochondria. Additionally, in a competitive binding assay, a three-fold molar excess of p104_554–593_ was required to obtain 50% inhibition of EB1 binding to recombinant p104 (data not shown), suggesting that short soluble fragments of p104 might not be good competitors for EB1-p104 binding. An alternative explanation could be that EB1 binding sites other than the one contained in p104 are available at the parasite surface. In this context, it is of interest that another +TIP, CLASP1 (CLIP-170 associating protein 1), which contains two SxIP motifs and acts as a verified EB1 interaction partner [27], was recently found decorating the entire surface of the schizont (unpublished observation from our laboratory). It is thus conceivable that SxIP motifs of parasite-associated CLASP1 provide additional EB1 docking sites. Considering the importance of MT recruitment for parasite persistence, the existence of redundant EB1 recruitment mechanisms at the parasite surface would not be surprising.

The schizont, as a cytoplasm-dwelling organism, shares to some extent properties with cellular organelles like the endoplasmic reticulum, the Golgi apparatus and the endosomes/lysosomes, which all depend on MT dynamics for their positioning and function in the cell (reviewed in [52]). p104 could fulfill a role similar to STIM1 (stromal-interaction molecule 1), one of the few well-characterized EB1-binding +TIPs described up till now that is not cytoplasmic. STIM1 is involved in ER growth and remodeling [53] and, by binding EB1, mediates interaction between MT ends and ER membranes, required for ER tubule extension and membrane remodeling [53].

To our knowledge, *Theileria* p104 provides the first example for a pathogen-derived protein capable of interacting with EB1 via a functional consensus SxIP motif. The movement protein of tobacco mosaic virus (TMV-MP), which facilitates cell-to-cell spread of infection, was also reported to interact with EB1 in vivo and in vitro, [54]. TMV-MP does not contain an SxIP motif, however, and also the mode of interaction with EB1 was not elucidated.

In summary, *Theileria* parasites have evolved mechanisms to ‘hijack’ proteins that function as ‘hubs’ (those involved in many interactions) or ‘bottlenecks’ (proteins central to many pathways) [55]. Plk1, an important regulator of mitosis [5] or IKK, a central regulator of NF-κB pathways [56], are two striking examples of important proteins recruited to the parasite surface. As a cytoplasmic parasite co-evolving with its mammalian host, the parasite has also acquired the ability to hijack EB1, the ‘master regulator’ of host cell MT dynamics, thereby gaining access to the regulatory mechanisms that control the microtubular cytoskeleton.

## Materials and Methods

### Screening of a *T. annulata* cDNA expression library with 1C12 antibody

A SMART cDNA library that consists of *T. annulata* cDNA embedded in the multiple cloning site of the phage λTriplEx2 (Clontech) was provided by Gordon Langsley [31]. *E. coli* were transduced with the phage library, and approximately 2 x 10^5^ plaques were screened with the mAb 1C12 [34] Phage DNA was converted into a plasmid (pTriplEx2) by site-specific recombination, and sequenced to identify *TA08425*.

### Cell culture, transfections and drug treatments

*T. annulata* (TaC12)-infected macrophages, the SV40-transformed cell line of *Theileria*-uninfected bovine macrophages (BoMac) and COS-7 cells were cultured as described previously [5]. Schizonts were purified from TaC12 cells, basically as described previously [57]. Transfections were performed following manufacturer's instructions using Lipofectamine 2000 (Invitrogen) for COS-7, or Amaxa 4D Nucleofection (Lonza) (cell solution SF, program DS103) for TaC12 cells. For depolymerization of MTs and the induction of cell cycle arrest in prometaphase, cells were treated with 0.1 µg/ml nocodazole (Biotrend) for 16 h, and harvested by shake-off. To arrest cells in metaphase, cells were harvested by shake-off following nocodazole treatment, and transferred into medium containing 20 µM MG132 (Alexis) for 2 h. For inhibition of Cdk1 activity, metaphase-synchronized TaC12 cells were incubated with 10 µM RO-3306 (Alexis) in MG132-containing media for 30 min.

### Generation of expression constructs

C-terminal GFP-tagged mouse EB1 and human EB3 were generous gifts from Anna Akhmanova (Utrecht University). The EB1-GFP plasmid was used as a template for subsequent cloning of mouse EB1 cDNA fragments into pmaxFP-Green N or Green C vectors (Lonza) by PCR and using *Sac*1 and *Kpn*1 restriction enzymes, or pEF6-Myc-His (invitrogen) with *Kpn*1 and *Not*1. For a list of primers, please see Table S1. For expression of V5-tagged proteins in COS-7 cells, coding regions of *TA08425* (*p104*) were amplified from genomic DNA (gDNA) from *Theileria annulata* (TaC12)-infected macrophages, and cloned using *Eco*RV and *Xho*1 into a modified version of pmaxCloning vector (Lonza) that contains a C-terminal V5 tag. Fragments of *TA17375* and *TA20980* were amplified from TaC12 cDNA and gDNA respectively, and *TA17545* was amplified from a cDNA clone (a kind gift from Gordon Langsley) prior to cloning into the modified pmax-V5 vector with *Eco*RV and *Xho*1. For mitochondrial targeting of p104, the 20 amino-acid C-terminal tail of the vaccinia virus F1L protein [46] was cloned downstream of the V5 tag in the pmaxCloning vector using *Age*1 and *Sac*1, prior to the subsequent insertion of *p104* fragments. The SKIP motif of p104 was mutated to SKNN by PCR mutagenesis. For live cell imaging, *p104* fragments were cloned into pmaxFP-Green C using *Hin*dIII and *Bam*H1. For expression of recombinant protein, *p104* fragments with a C-terminal V5 tag were cloned into the pFN18A HaloTag T7 Flexi vector (Promega), using *Sgf*1 and *Nco*1. For expression of recombinant mouse EB1 in *E. coli*, mouse EB1 cDNA was cloned with a C-terminal myc tag into the pFN18A HaloTag T7 Flexi vector using *Sgf*1 and *Nco*1 restriction enzymes, or the pGEX-6P2 vector with *Eco*R1 and *Not*1. *T. annulata* EB1 was amplified from TaC12 cDNA and cloned using *Nco*1 and *Kpn*1 into the expression vector pHIS-parallel1 [58]. For expression of recombinant GFP-p104 fragments, the copGFP coding region was amplified from pmaxFP-Green N vector and cloned into the pGEX-6P2 vector (GE healthcare) with *Bam*H1 and *Eco*R1, before subsequent insertion of fragments of *p104* with *Eco*R1 and *Xho*1.

### Expression of recombinant protein, pull-down assays and Western blotting

TaC12, BoMAC, and COS-7 lysates were prepared for western blotting and pull-down assays in modified RIPA buffer (50 mM Tris-HCl pH7.4, 150 mM NaCl, 1 mM EDTA. 1% NP-40, 0.25% Na-deoxycholate, 2 mM Na-vanadate, 25 mM NaF, protease inhibitor cocktail, Roche, and phosphatase inhibitor cocktail, Sigma). For removal of phosphate groups prior to SDS-PAGE analysis, lysates were prepared in the absence of phosphatase inhibitors and treated with lambda protein phosphatase (NEB), following manufacturer's instructions. Recombinant p104-V5 and EB1-myc protein were expressed and purified from *E. coli* using the HaloTag Protein Purification System (Promega) following manufacturer's instructions. For pull-down assays with recombinant p104, approximately 500 µg COS-7 lysate were incubated with Halo-tagged proteins overnight, before being washed 5 times in wash buffer (containing 500 mM NaCl and 0.01% tween-20), and eluted by TEV protease cleavage. Recombinant mouse EB1 and GFP-p104_554–593_ were purified from *E. coli* using Glutathione Sepharose 4b (GE Healthcare) followed by cleavage with Precision protease (GE Healthcare), following manufacturer's instructions. For pull-down assays with GST tagged EB1, 1 mg TaC12 lysate was incubated with GST-EB1 coated Glutathione Sepharose 4b for 3 hours, before being washed 5 times in wash buffer (containing 500 mM NaCl and 0.01% tween-20), and eluted by precision protease cleavage. Primary antibodies used for western blotting and immunofluorescence analysis were mouse mAbs anti-p104 (clone 1C12), anti-V5 (Invitrogen), anti-α-tubulin (clone DM1A, Sigma), anti-cyclin B1 (clone GNS-11, Pharmingen), c-Myc (9E10, Santa Cruz), anti-His (GE Healthcare) rat monoclonal anti-EB1 (clone KT51 Absea), and rabbit polyclonal anti-Turbo GFP (AB514, Evrogen). Rabbit polyclonal anti-TaSP antibodies were generated by the laboratory of Isabel Roditi (Bern) using the procedure described by Schnittger et al. [59].

### Immunofluorescence analysis, time-lapse imaging and microinjection

Interphase cells were grown on coverslips, and cells harvested by mitotic shake-off were seeded on poly-L-lysine coated coverslips (Sigma). Samples were fixed with methanol for 10 min at −20°C or with 4% paraformaldehyde in PBS for 10 min at room temperature followed by permeabilization in 0.2% Triton-X-100. Antibody incubations were performed in PBS containing 10% heat-inactivated FCS. DNA was stained with DAPI, and the mitochondria with MitoTracker (Molecular Probes), and cells were mounted using DAKO mounting media. Wide-field microscopy was performed with a Nikon Eclipse 80i microscope equipped with a Retiga 2000R CCD camera (Qimaging) using 60x and 100x Plan Apo objectives (Nikon) and Openlab 5 software (Improvision). Images were processed using Photoshop (Adobe). Time-lapse imaging was performed by recording fluorescence at 2 sec intervals for 2 min using a TE2000E-PFS microscope (Nikon) equipped with a Plan Fluor 60x objective (Nikon), Orca ER CCD camera (Hamamatsu), and incubation chamber (Life Imaging Services). Microinjection was performed using the FemtoJet microinjection system and Femtotip II microinjection capillaries (Eppendorf).

### Bioinformatics searches

A schematic presentation of the two approaches that were followed is shown in Figure S5. For the initial identification of *p104*, *T. annulata* GeneDB (http://old.genedb.org/genedb/annulata) was queried using the option ‘Complex/Boolean Query’ for genes encoding ‘proteins containing a predicted signal peptide’ and ‘proteins containing a predicted GPI-anchor’ yielding 559 and 33 entries, respectively. Intersection of the selected queries resulted in 19 candidates. Downloaded sequences were screened for the presence of an SxIP motif (LIG_SxIP_EBH_1) using the ELM (Eukaryotic Linear Motif) resource (http://elm.eu.org) and for representation in the *T. annulata* schizont proteome database [29]. *TA08425* (*p104*) emerged as the only candidate fulfilling all criteria: signal peptide, GPI-anchor, evidence of protein expression and an SxIP motif located in a disordered region and surrounded by the appropriate residues as defined [28], [33].

The following approach was used to search for SxIP motif-containing proteins in *T. annulata*, *T. parva* and the non-transforming *T. orientalis*: a genome-wide search at predicted proteome-level in all three publicly available *Theileria* genomes was performed. *T. parva* and *T. annulata* proteome sequences were obtained from PiroplasmaDB 2.0 release while the *T. orientalis* sequence was retrieved from the NCBI genome database. These consisted of 4129, 3799 and 3734 predicted proteins, respectively. We obtained the SxIP motif pattern (LIG_SxIP_EBH_1) from the Eukaryotic Linear Motif (ELM) database [60]. To screen the full proteome, we used a simple SxIP motif search using the EMBOSS (http://emboss.sourceforge.net/) ‘preg’ utility. The filtered proteins were submitted through a perl script to the ELM web server for the prediction of SxIP motif with disorderness and required surrounding residues. Signal peptides were predicted for SxIP proteins using standalone SignalP 3.0 [61] and SignalP 4.0 [62] tools with default settings. For GPI anchor prediction, the PredGPI tool [63] with default parameters was used. To establish the species specificity, we performed the ortholog analysis using OrthoMCL 2.0 [64]. For OrthoMCL analysis we included 41 species. For *Apicomplexa* we included *Babesia*, *Plasmodium*, *Toxoplasma*, *Cryptosporidium*, *Eimeria* and *Neospora* species. In addition to *Theileria* and other apicomplexan species, to determine phyletic distribution amongst other Eukaryotes, we included a major ciliate, *Perkinsus*, a couple of *Chromerids*, Green, Brown, Red algal genomes, *Arabidopsis*, Diatoms, etc (for details, please see ‘Taxonomy list’ in Table S3). The OrthoMCL clusters were generated on default settings. Based on the phyletic distribution of Ortholog clusters as determined by Ortho-MCL, we categorized gene clusters as restricted to *Theileria* (termed as ‘Theileria-specific’, or restricted to apicomplexan species (referred to as ‘Apicomplexa-specific’) or commonly present across Eukaryotes (referred to as ‘Eukaryotes’).

## Supporting Information

Figure S1 **Sequence analysis of** ***T. annulata*** **p104 (TA08425) expressed in TaC12 cells.** Predicted aa sequence of *T. annulata p104* obtained from TaC12 cells (Accession number GenBank JX965955). Predicted signal peptide sequence and GPI anchor sequences are underlined. FAINT domains (InterPro domain DUF529, IPR007480) are highlighted in yellow, and regions of low complexity are highlighted in grey. The SKIP motif is highlighted in red.
(TIF)

Figure S2 **The SxIP motif-containing proteins TA17375, TA20980 and TA17545 do not co-localize with endogenous EB1 at MT plus ends.** (A). Image of a COS-7 cell transiently expressing p104-CT-V5 (as a positive control), fixed with ice-cold methanol and stained with anti-EB1 (red) and anti-V5 (green); the white rectangle indicates the magnified cytoplasmic region. DNA is stained with DAPI (blue). Scale bar = 10 µm. (B). A 312 aa fragment of TA17375 encompassing a putative EB1-binding motif (KTTFIPNNG) fails to co-localize with EB1 at MT plus ends. (C). The C-terminal fragment of TA20980 (aa 603–989) encompassing a putative EB1-binding motif (RPSKIPIKQ) and two basically charged nuclear localization signals (NLS) (KKKKIK and PKKRRRP) fails to co-localize with EB1 at MT plus ends and is detected in the nucleus of COS-7 cells. (D). The N-terminal fragment of TA20980 (aa 21–513) encompassing a putative EB1-binding motif (KPSPIPKPR) and three NLS (KKRKKV, KKKKPK, PKRTKK) fails to co-localize with EB1 at MT plus ends and is detected in the nucleus of COS-7 cells. (E). TA17545, encompassing a putative EB1-binding motif (KPSKIPVHV) and a basically charged NLS (QKKRIK) fails to co-localize with EB1 at MT plus ends and is detected in the nucleus of COS-7 cells and in the cytoplasm.
(TIF)

Figure S3 **EB3-GFP interacts with the schizont surface.** Image of a TaC12 cell expressing EB3-GFP. The schizont was stained using 1C12 (red). DNA is stained with DAPI (blue). Scale bar = 5 µm.
(TIF)

Figure S4 **The monoclonal antibody KT51 does not cross-react with** ***T. annulata*** **EB1.** (A). The KT51 antibody does not recognize recombinant *T. annulata* EB1. Lysates of *E. coli* expressing recombinant Halo-mEB1-myc (mouse EB1) or His-TaEB1 (*T. annulata* EB1) were subjected to SDS-PAGE followed by immunoblot analysis using anti-EB1 (rat monoclonal KT51) and anti-His antibodies. (B). The KT51 antibody does not recognize endogenous *T. annulata* EB1. Lysates were prepared from TaC12 cells, uninfected BoMAC cells or purified schizonts, and equal amount of lysates subjected to SDS-PAGE analysis. Immunoblot analysis with anti-EB1 (KT51) confirmed that this antibody does not recognize *T. annulata* EB1. Immunoblot analysis with 1C12 confirmed the presence of parasite proteins in the purified schizont sample, while immunoblot with anti-tubulin confirmed the absence of host cell tubulin in purified schizont preparations.
(TIF)

Figure S5 **Schematic presentation of bioinformatics searches for SxIP motif-containing proteins in** ***Theileria*** **.** Bioinformatic searches were conducted using two different approaches: Approach 1 aimed at identifying GPI-anchored *T. annulata* schizont proteins capable of binding EB1 via a consensus SxIP motif. A manual web-based bioinformatics search was performed in GeneDB (http://old.genedb.org/genedb/annulata/) which revealed 559, 33 and 19 genes encoding ‘proteins containing a predicted signal peptide’, ‘proteins containing a predicted GPI-anchor’ and intersection of both, respectively. In Approach 2, a detailed genome-wide bioinformatics screen of all three publicly available genomes of *Theileria* species was conducted. For further details, please see the Materials and Methods sections. Details of candidate genes identified in both approaches have been provided in Tables S2 and S3.
(PPTX)

Movie S1 **EB1-GFP tracks MT plus ends and labels the schizont surface in TaC12 cells.**
(MP4)

Movie S2 **GFP-p104-_521–634_ tracks MT plus ends in COS-7 cells.**
(MP4)

Movie S3 **GFP-p104-_554–593_ microinjected into TaC12 cells labels the centrosome, tracks MT plus ends and decorates the surface of the schizont.** The arrow-head shows the position of the parasite in the cell. The inlay represents a magnification of the schizont.
(MP4)

Table S1 **List of primers used for PCR amplification and generation of the different plasmid constructs as described in** **Materials and Methods** **.**
(DOCX)

Table S2 **Lists with Gene IDs of 559** ***T. annulata*** **genes encoding proteins containing a predicted signal peptide, 33** ***T. annulata*** **genes encoding proteins predicted to contain a GPI-anchor sequence, and 19 genes encoding proteins containing both.** For the latter, the annotation, the predicted expression stage (according to GeneDB) and mass are also provided.
(DOCX)

Table S3 **This Excel file contains the results of a comprehensive bioinformatics search for ‘SxIP motif’-containing proteins across the three available** ***Theileria*** **genomes (** ***T. annulata*** **,** ***T. parva*** **and** ***T. orientalis*** **); the Excel sheets are labeled accordingly.** SignalP 3.0 and SignalP 4.0 algorithms were used to predict the presence (S) or absence (NS) of signal peptides. PredGPI was used to predict the presence of a GPI anchor sequence. Based on the phyletic distribution of Ortholog clusters as determined by Ortho-MCL, gene clusters were categorized as restricted to *Theileria* (termed as ‘Theileria-specific’, or restricted to apicomplexan species (referred to as ‘Apicomplexa-specific’) or commonly present across Eukaryotes (referred to as ‘Eukaryotes’). The sheet labeled ‘Taxonomy list’ contains a list of genomes interrogated.
(XLS)
