# Supplementary figures and images for: Recruitment of EB1, a Master Regulator of Microtubule Dynamics, to the Surface of the Theileria annulata Schizont
Source: PLoS Pathog. 2013 May 9;9(5):e1003346. doi: 10.1371/journal.ppat.1003346 (PMC3649978; doi:10.1371/journal.ppat.1003346)

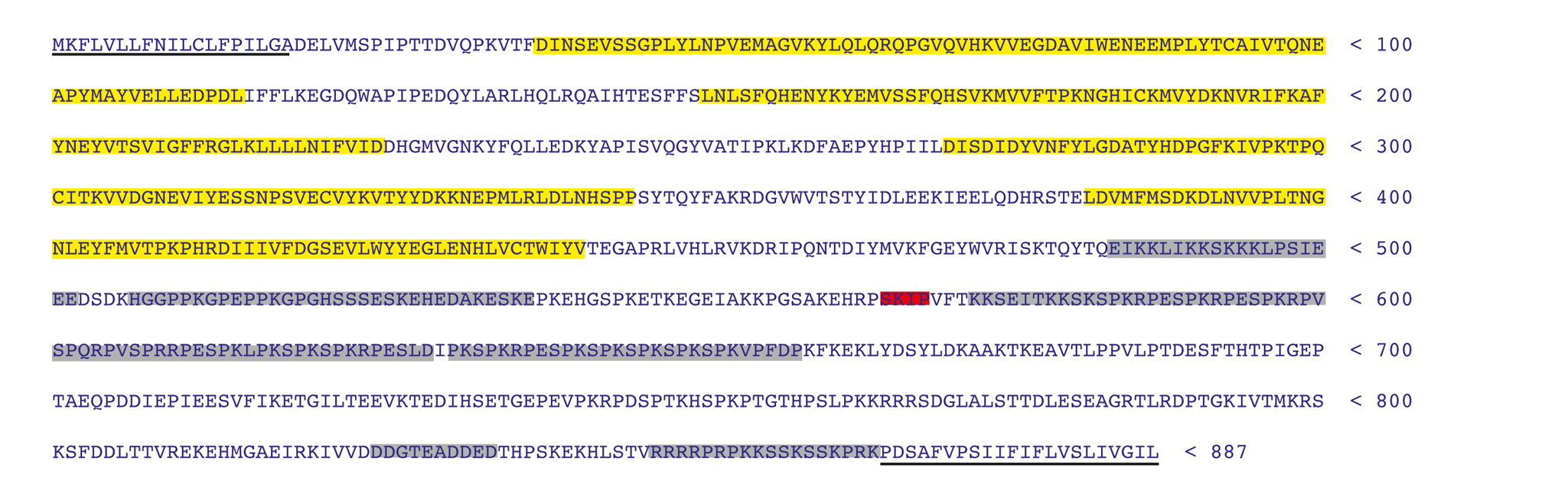

Supplement: Figure S1 — Sequence analysis of T. annulata p104 (TA08425) expressed in TaC12 cells. Predicted aa sequence of T. annulata p104 obtained from TaC12 cells (Accession number GenBank JX965955). Predicted signal peptide sequence and GPI anchor sequences are underlined. FAINT domains (InterPro domain DUF529, IPR007480) are highlighted in yellow, and regions of low complexity are highlighted in grey. The SKIP motif is highlighted in red. (TIF) [file ppat.1003346.s001.tif]

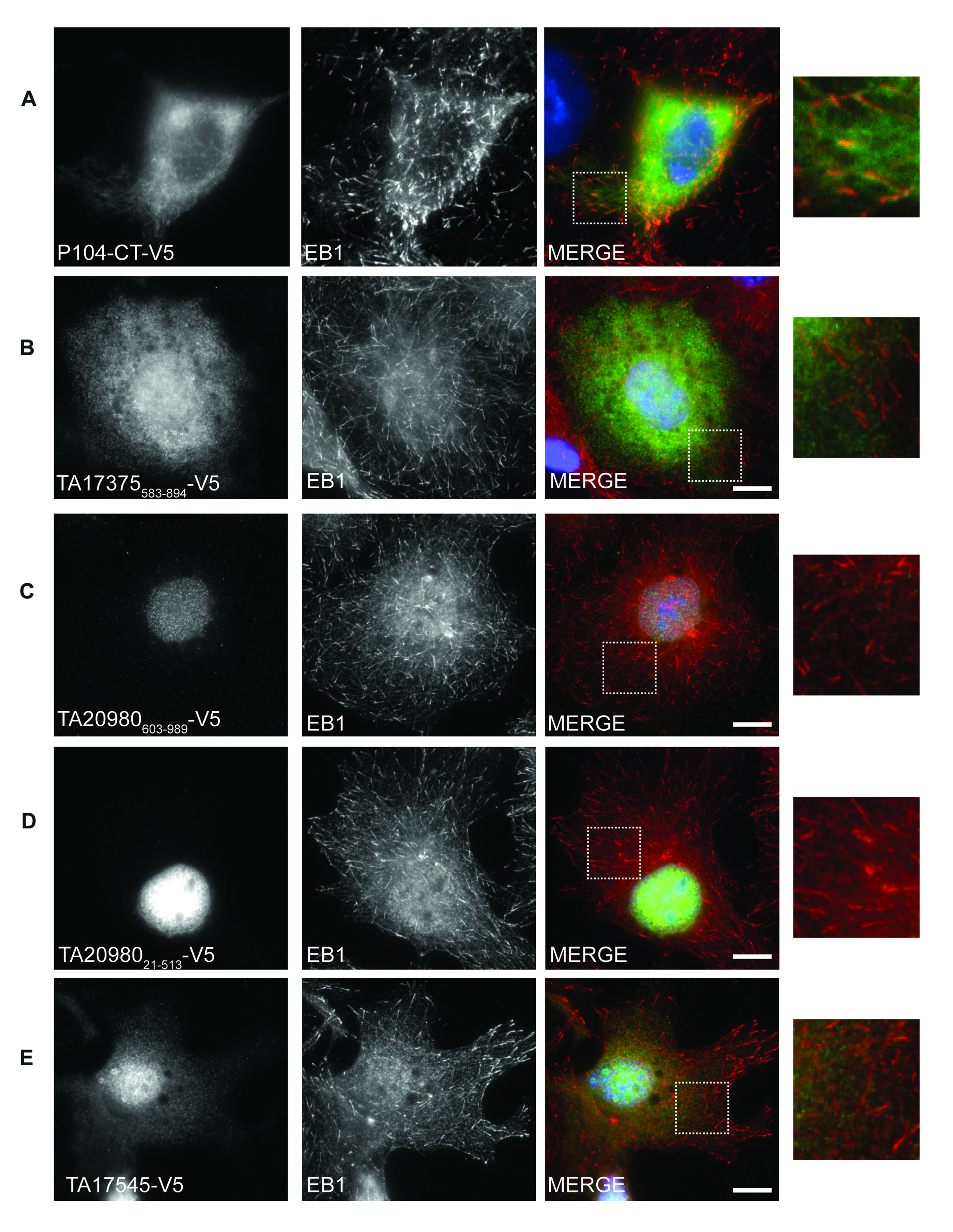

Supplement: Figure S2 — The SxIP motif-containing proteins TA17375, TA20980 and TA17545 do not co-localize with endogenous EB1 at MT plus ends. (A). Image of a COS-7 cell transiently expressing p104-CT-V5 (as a positive control), fixed with ice-cold methanol and stained with anti-EB1 (red) and anti-V5 (green); the white rectangle indicates the magnified cytoplasmic region. DNA is stained with DAPI (blue). Scale bar = 10 µm. (B). A 312 aa fragment of TA17375 encompassing a putative EB1-binding motif (KTTFIPNNG) fails to co-localize with EB1 at MT plus ends. (C). The C-terminal fragment of TA20980 (aa 603–989) encompassing a putative EB1-binding motif (RPSKIPIKQ) and two basically charged nuclear localization signals (NLS) (KKKKIK and PKKRRRP) fails to co-localize with EB1 at MT plus ends and is detected in the nucleus of COS-7 cells. (D). The N-terminal fragment of TA20980 (aa 21–513) encompassing a putative EB1-binding motif (KPSPIPKPR) and three NLS (KKRKKV, KKKKPK, PKRTKK) fails to co-localize with EB1 at MT plus ends and is detected in the nucleus of COS-7 cells. (E). TA17545, encompassing a putative EB1-binding motif (KPSKIPVHV) and a basically charged NLS (QKKRIK) fails to co-localize with EB1 at MT plus ends and is detected in the nucleus of COS-7 cells and in the cytoplasm. (TIF) [file ppat.1003346.s002.tif]

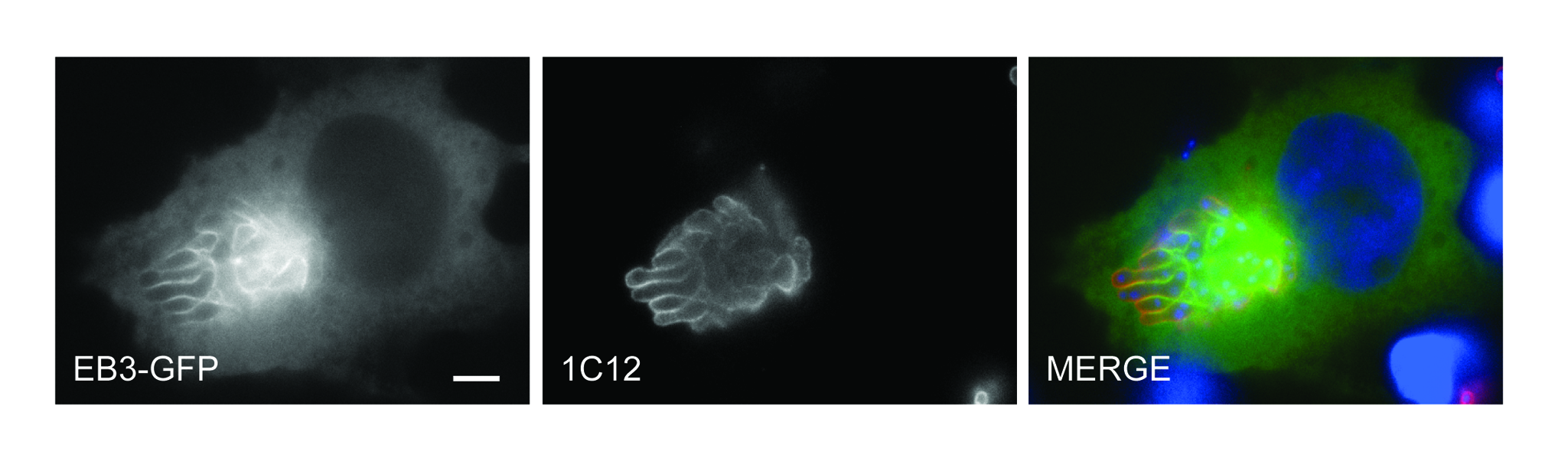

Supplement: Figure S3 — EB3-GFP interacts with the schizont surface. Image of a TaC12 cell expressing EB3-GFP. The schizont was stained using 1C12 (red). DNA is stained with DAPI (blue). Scale bar = 5 µm. (TIF) [file ppat.1003346.s003.tif]

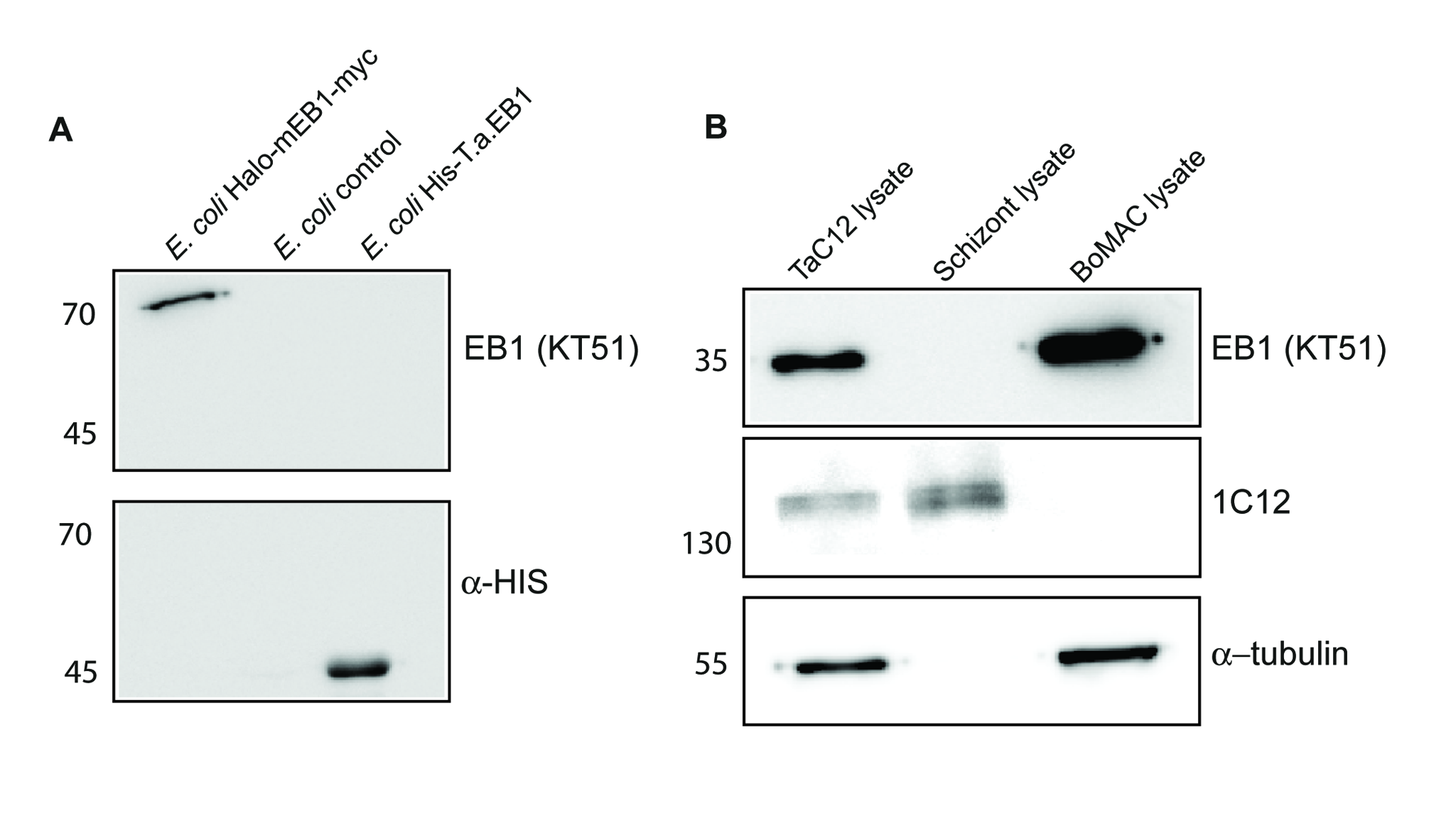

Supplement: Figure S4 — The monoclonal antibody KT51 does not cross-react with T. annulata EB1. (A). The KT51 antibody does not recognize recombinant T. annulata EB1. Lysates of E. coli expressing recombinant Halo-mEB1-myc (mouse EB1) or His-TaEB1 (T. annulata EB1) were subjected to SDS-PAGE followed by immunoblot analysis using anti-EB1 (rat monoclonal KT51) and anti-His antibodies. (B). The KT51 antibody does not recognize endogenous T. annulata EB1. Lysates were prepared from TaC12 cells, uninfected BoMAC cells or purified schizonts, and equal amount of lysates subjected to SDS-PAGE analysis. Immunoblot analysis with anti-EB1 (KT51) confirmed that this antibody does not recognize T. annulata EB1. Immunoblot analysis with 1C12 confirmed the presence of parasite proteins in the purified schizont sample, while immunoblot with anti-tubulin confirmed the absence of host cell tubulin in purified schizont preparations. (TIF) [file ppat.1003346.s004.tif]
