# Supplementary material for: Recruitment of EB1, a Master Regulator of Microtubule Dynamics, to the Surface of the Theileria annulata Schizont
Source: PLoS Pathog. 2013 May 9;9(5):e1003346. doi: 10.1371/journal.ppat.1003346 (PMC3649978; doi:10.1371/journal.ppat.1003346)
Supplement: Table S1 — List of primers used for PCR amplification and generation of the different plasmid constructs as described in Materials and Methods . (DOCX) [file ppat.1003346.s009.docx]

Supplementary Materials. List of primers used. The target plasmid is listed, and the restriction sites used for cloning (described in the text) are underlined. (F = forward; R = reverse)

| **Gene fragment** | **Target plasmid** |  | **Primer sequence 5’-3’** |
| --- | --- | --- | --- |
| P104_470-871_ | Pmax-V5 | F | CGGATATCATGGGTGAGTATTGGGTTAGG |
| P104_470-871_ | Pmax-V5 | R | CGCTCGAGTTTCCTTGGTTTTGATGAC |
| P104_20-469_ | Pmax-V5 | F | CGGATATCATGGACGAGCTGGTTATGAGTC |
| P104_20-469_ | Pmax-V5 | R | CGCTCGAGAAACTTGACCATGTATATATC |
|  | SKIP-SKNN mutation | F | CACAGGCCAAGCAAGAATAACGTATTCACCAAGAAA |
|  | SKIP-SKNN mutation | R | TTTCTTGGTGAATACGTTATTCTTGCTTGGCCTGTG |
| Vaccinia virus F1L C-term tail | Pmax-V5 | F | GCGCACCGGTAAGCTTATTGGCATCACAGCTATCATGTTTGCTACATATAAAACTCTCAAATACATGATAGGATGAGTTTAAACGCGC |
| Vaccinia virus F1L C-term tail | Pmax-V5 | R | GCGCGTTTAAACTCATCCTATCATGTATTTGAGAGTTTTATATGTAGCAAACATGATAGCTGTGATGCCAATAAGCTTACCGGTGCGC |
| P104_470-871_-V5 | pFN18A HaloTag T7 Flexi vector | F | ATATATGCGATCGCCGGTGAGTATTGGGTTAGGATT |
| P104_470-871_-V5 | pFN18A HaloTag T7 Flexi vector | R | TATATAGTTTAAACCGTAGAATCGAGACCGAGGAGAGGGTTAGGGATAGGCTTACCTTTCCTTGGTTTTGATGACTTTGATGACTT |
| P104_20-469-_V5 | pFN18A HaloTag T7 Flexi vector | F | ATATATGCGATCGCCGACGAGCTGGTTATGAGTCC |
| P104_20-469_-V5 | pFN18A HaloTag T7 Flexi vector | R | ATATATGTTTAAACCGTAGAATCGAGACCGAGGAGAGGGTTAGGGATAGGCTTACCAAACTTGACCATGTATATATCTGTATTTTG |
| P104_521-634_ | pmaxFP-GreenN | F | CGAAGCTTACCATGccagggcattcttcttca |
| P104_521-634_ | pmaxFP-GreenN | R | CGGGATCCCGTGATTTTGGTATGTCGAG |
| P104_521-634_ | Pmax-V5 | F | ATATGATATCATGCCAGGGCATTCTTCTTCAG |
| P104_521-634_ | Pmax-V5 | R | ATATCTCGAGAGATTTTGGTATGTCGAG |
| p104_554_ | pGEX-6P2 copGFP | F | ATATGAATTCAGCTAAAAAACCTGGGTC |
| p104_554_ | pGEX-6P2 copGFP | R | ATATCTCGAGACTTATGGTCTTTTGGGTGAC |
| copGFP | pGEX-6P2 | F | ATATGGATCCATGGAGAGCGACGAGAGC |
| copGFP | pGEX-6P2 | R | ATATGAATTCGGTTCTTCACCGGCATCTGC |
| TA17375_583-894_ | Pmax-V5 | F | ATATGATATCATGGGCAAATATACTTTATGG- |
| TA17375_583-894_ | Pmax-V5 | R | ATATCTCGAGAAAACCATTAGAACTCAAAG |
| TA20980_603-989_ | Pmax-V5 | F | ATATGATATCATGTCCACGCCAAATACTGTTAAAC |
| TA20980_603-989_ | Pmax-V5 | R | ATATCTCGAGTTTGCTATTCCTTCTATTTC |
| TA20980_21-513_ | Pmax-V5 | F | ATATGATATCATGGATAAAGATCCACAGAAGG |
| TA20980_21-513_ | Pmax-V5 | R | ATATCTCGAGGTCTTCTTCTGATTCTTG |
| TA17545 | Pmax-V5 | F | ATATGATATCATGGCTGATCAACCAAATGATA |
| TA17545 | Pmax-V5 | F | ATATCTCGAGTTCATCTTTACTACGTCTTC |
| mEB1 | pEF6-Myc-His | F | ATGGTACCATGGCAGTGAATGTGTAC |
| mEB1 | pEF6-Myc-His | R | ATGCGGCCGCCATACTCTTCTTGTTCCTC |
| mEB1 | pmaxFP-GreenN | F | GCGAGCTCATGGCAGTGAATGTGTACTC |
| mEB1_1-267_ | pmaxFP-GreenN | R | GCGGTACCTCCTCTTCTTGTTCCTCCTGTG |
| mEB1_1-133_ | pmaxFP-GreenN | R | GCGGTACCTCTTGACCTTGACCTTGTCTG |
| mEB1_1-133_ | pmaxFP-GreenC | F | TCGAGCTCACATGGCAGTGAATGTGTACTC |
| mEB1_125-268_ | pmaxFP-GreenN | F | GCGAGCTCATGGATCCTGTAGCTGCCAG |
| mEB1_125-268_ | pmaxFP-GreenN | R | GCGGTACCTCATACTCTTCTTGTTCTTCC |
| mEB1_208-268_ | pmaxFP-GreenN | F | GCGAGCTCATGGAAGACTTGGAGAAGGAGAG |
| mEB1_208-268_ | pmaxFP-GreenC | F | TCGAGCTCGAATGGAAGACTTGGAGAAGGA |
| mEB1_208-268_ | pmaxFP-GreenC | R | GCGGTACCTTAATACTCTTCTTGTTCCT |
| mEB1_208-251_ | pmaxFP-GreenN | R | GCGGTACCTCTTCATCTGTGGCATAAAGAAT |
| mEB1_208-251_ | pmaxFP-GreenC | R | GCGGTACCTTATTCATCTGTGGCATAAA |
| mEB1 | pGEX-6P2 | F | ATATGAATTCCCATGGCAGTGAATGTGTAC |
| mEB1 | pGEX-6P2 | R | ATGCGGCCGCCATACTCTTCTTGTTCCTC |
| mEB1 | pFN18A HaloTag T7 Flexi vector | F | ATATGCGATCGCCATGGCAGTGAATGTGTAC |
| mEB1 | pFN18A HaloTag T7 Flexi vector | R | ATATGTTTAACCCAGATCCTCTTCTGAGATG |
| *T. annulata* EB1 | pHis-parallel1 | F | ATATCCATGGCAGACAAAGGCCAATTTAATGG |
| *T. annulata* EB1 | pHis-parallel1 | R | ATATGGTACCTTAATTATTAGCGTACATG |
