# Supplementary material for: Recruitment of EB1, a Master Regulator of Microtubule Dynamics, to the Surface of the Theileria annulata Schizont
Source: PLoS Pathog. 2013 May 9;9(5):e1003346. doi: 10.1371/journal.ppat.1003346 (PMC3649978; doi:10.1371/journal.ppat.1003346)
Supplement: Table S2 — Lists with Gene IDs of 559 T. annulata genes encoding proteins containing a predicted signal peptide, 33 T. annulata genes encoding proteins predicted to contain a GPI-anchor sequence, and 19 genes encoding proteins containing both. For the latter, the annotation, the predicted expression stage (according to GeneDB) and mass are also provided. (DOCX) [file ppat.1003346.s010.docx]

**Supplemental tables 2**

Please note that the version of GeneDB used for this analysis (old.dgnedb.org) [[1](#_ENREF_1)] will be taken offline at the end of April 2013. An analysis using the algorithms used in the new improved GeneDB ([www.genedb.org](http://www.genedb.org)) [[2](#_ENREF_2)] will give partly different results.

**Signal peptide**

List of 559 *T. annulata* proteins extracted from the *Theileria annulata* GeneDB database [[3](#_ENREF_3)] (<http://old.genedb.org/genedb/annulata/>) GeneDB website Version 2.1, using Complex/Boolean Query setting: 'Proteins containing a predicted signal peptide'

(algorithm used in this version of geneDB: Signal peptide predictions by SignalP 2.0)

TA02480

TA02485

TA02510

TA02580

TA02585

TA02590

TA02620

TA02700

TA02710

TA02715

TA02725

TA02740

TA02770

TA02785

TA02935

TA02960

TA03110

TA03115

TA03120

TA03140

TA03145

TA03150

TA03155

TA03160

TA03165

TA03175

TA03210

TA03300

TA03305

TA03310

TA03350

TA03475

TA03530

TA03540

TA03545

TA03590

TA03600

TA03615

TA03620

TA03640

TA03670

TA03680

TA03735

TA03740

TA03745

TA03750

TA03755

TA03770

TA03775

TA03850

TA03855

TA03870

TA03910

TA03950

TA03995

TA04105

TA04155

TA04225

TA04375

TA04390

TA04430

TA04450

TA04545

TA04635

TA04670

TA04675

TA04680

TA04685

TA04690

TA04695

TA04705

TA04785

TA04795

TA04825

TA04830

TA04925

TA04935

TA04950

TA04960

TA05000

TA05015

TA05025

TA05070

TA05145

TA05175

TA05240

TA05255

TA05315

TA05320

TA05375

TA05390

TA05440

TA05495

TA05510

TA05530

TA05540

TA05550

TA05555

TA05560

TA05565

TA05570

TA05615

TA05705

TA05735

TA05750

TA05760

TA05775

TA05815

TA05820

TA05825

TA05860

TA05870

TA05965

TA05985

TA06030

TA06050

TA06065

TA06075

TA06115

TA06220

TA06280

TA06320

TA06355

TA06360

TA06375

TA06380

TA06420

TA06470

TA06510

TA06560

TA06595

TA06620

TA06675

TA06680

TA06740

TA06775

TA06810

TA06875

TA06945

TA07070

TA07085

TA07095

TA07130

TA07145

TA07200

TA07205

TA07210

TA07230

TA07270

TA07415

TA07455

TA07465

TA07470

TA07480

TA07505

TA07535

TA07565

TA07585

TA07595

TA07630

TA07865

TA07870

TA08105

TA08110

TA08235

TA08260

TA08325

TA08360

TA08365

TA08370

TA08415

TA08425

TA08445

TA08485

TA08609

TA08620

TA08635

TA08670

TA08690

TA08715

TA08745

TA08880

TA08935

TA09050

TA09080

TA09115

TA09140

TA09255

TA09265

TA09320

TA09330

TA09365

TA09425

TA09430

TA09440

TA09445

TA09450

TA09460

TA09465

TA09470

TA09475

TA09480

TA09485

TA09490

TA09495

TA09500

TA09520

TA09660

TA09670

TA09675

TA09720

TA09755

TA09756

TA09760

TA09770

TA09775

TA09785

TA09790

TA09795

TA09800

TA09805

TA09810

TA09865

TA09905

TA09975

TA10055

TA10060

TA10110

TA10230

TA10275

TA10285

TA10310

TA10315

TA10450

TA10480

TA10500

TA10510

TA10645

TA10705

TA10720

TA10740

TA10755

TA10825

TA10865

TA10880

TA10925

TA10955

TA11005

TA11015

TA11020

TA11050

TA11230

TA11325

TA11360

TA11365

TA11385

TA11390

TA11400

TA11410

TA11420

TA11465

TA11480

TA11485

TA11495

TA11565

TA11575

TA11680

TA11685

TA11755

TA11770

TA11805

TA11890

TA11905

TA11915

TA11935

TA11950

TA11955

TA11965

TA12005

TA12035

TA12045

TA12075

TA12165

TA12185

TA12215

TA12225

TA12230

TA12370

TA12420

TA12465

TA12590

TA12630

TA12665

TA12705

TA12720

TA12850

TA13045

TA13075

TA13185

TA13245

TA13360

TA13370

TA13425

TA13435

TA13480

TA13495

TA13505

TA13530

TA13590

TA13720

TA13750

TA13755

TA13785

TA13810

TA13815

TA13825

TA13845

TA13885

TA13930

TA13955

TA13970

TA13980

TA14030

TA14085

TA14095

TA14135

TA14200

TA14205

TA14210

TA14215

TA14250

TA14270

TA14275

TA14290

TA14295

TA14310

TA14455

TA14465

TA14490

TA14545

TA14665

TA14675

TA14690

TA14765

TA14780

TA14945

TA14955

TA14985

TA15055

TA15150

TA15195

TA15265

TA15285

TA15305

TA15340

TA15415

TA15450

TA15505

TA15540

TA15570

TA15650

TA15665

TA15705

TA15715

TA15745

TA15760

TA15910

TA15960

TA16005

TA16010

TA16020

TA16025

TA16030

TA16040

TA16045

TA16050

TA16055

TA16125

TA16145

TA16160

TA16190

TA16195

TA16200

TA16205

TA16215

TA16220

TA16250

TA16270

TA16420

TA16520

TA16525

TA16565

TA16580

TA16680

TA16685

TA16735

TA16750

TA16780

TA16820

TA16830

TA16860

TA16880

TA16895

TA17050

TA17055

TA17105

TA17110

TA17115

TA17120

TA17125

TA17130

TA17175

TA17180

TA17200

TA17210

TA17215

TA17220

TA17265

TA17270

TA17305

TA17315

TA17325

TA17340

TA17355

TA17375

TA17405

TA17410

TA17425

TA17445

TA17450

TA17475

TA17480

TA17485

TA17505

TA17535

TA17540

TA17545

TA17550

TA17555

TA17560

TA17575

TA17585

TA17600

TA17610

TA17635

TA17655

TA17670

TA17695

TA17705

TA17740

TA17745

TA17885

TA17945

TA17955

TA18060

TA18070

TA18080

TA18155

TA18200

TA18210

TA18295

TA18300

TA18363

TA18420

TA18450

TA18500

TA18505

TA18535

TA18540

TA18545

TA18625

TA18700

TA18745

TA18750

TA18755

TA18760

TA18765

TA18770

TA18835

TA18855

TA18860

TA18865

TA18870

TA18880

TA18885

TA18965

TA18990

TA19005

TA19045

TA19075

TA19115

TA19170

TA19195

TA19230

TA19235

TA19245

TA19255

TA19275

TA19280

TA19320

TA19360

TA19365

TA19440

TA19475

TA19600

TA19635

TA19650

TA19670

TA19685

TA19760

TA19830

TA19865

TA19885

TA19905

TA19930

TA20040

TA20082

TA20083

TA20085

TA20090

TA20150

TA20215

TA20260

TA20310

TA20315

TA20325

TA20430

TA20435

TA20440

TA20505

TA20520

TA20560

TA20600

TA20615

TA20640

TA20675

TA20781

TA20790

TA20855

TA20870

TA20890

TA20930

TA20980

TA20985

TA20990

TA21005

TA21040

TA21045

TA21120

TA21205

TA21230

TA21260

TA21270

TA21280

TA21370

TA21450

TA21470

TA21510

TA21515

**GPI-anchor**

List of 33 *T. annulata* proteins extracted from the *Theileria annulata* GeneDB database (<http://old.genedb.org/genedb/annulata/>) GeneDB website Version 2.1, using Complex/Boolean Query setting: 'Proteins containing a predicted GPI anchor'

(algorithm used in this version of geneDB: GPI-anchor prediction by DGPI v2.04)

TA02585

TA02590

TA03755

TA04100

TA04565

TA04785

TA04825

TA05320

TA06150

TA06195

TA06475

TA06510

TA06940

TA07480

TA07970

TA08425

TA09660

TA09756

TA10740

TA13720

TA13775

TA13820

TA14310

TA15490

TA16100

TA16565

TA17050

TA17210

TA17220

TA18965

TA18995

TA19445

TA21430

**Signal peptide and GPI anchor**

Query results (Signal peptide and GPI-anchor) were combined using the setting 'Intersect' which identifies common results between the two files. This resulted in a list of 19 *T. annulata* proteins.

Sp: mRNA detected in sporozoites; MS: mRNA detected in macroschizont; Me: mRNA detected in merozoites; Pi: mRNA detected in piroplasm.

| **Identifier** | **Description** | **Expressed at stage** | **Mass** |
| --- | --- | --- | --- |
| TA05320 | Hypothetical protein |  | (20.2 kDa) |
| TA09756 | Hypothetical protein (FAINT) | MS | (19.3 kDa) |
| TA14310 | Hypothetical protein |  | (19.1 kDa) |
| TA16565 | Hypothetical protein |  | (26.2 kDa) |
| TA08425 | p104, putative *Theileria parva* microneme-rhoptry antigen | MS, Me | (101.9 kDa) |
| TA04785 | Tpr-related protein family member, putative |  | 21.4 |
| TA02590 | Hypothetical protein | MS | 42.3 kDa |
| TA02585 | Hypothetical protein |  | 20.1 kDa |
| TA07480 | Hypothetical protein | MS | 56.3 kDa |
| TA09660 | Putative vesicle transport protein | MS | 24.0 kDa |
| TA04825 | Hypothetical protein |  | 123.0 kDa |
| TA17050 | Merozoite-piroplasm surface antigen Tams1[[4](#_ENREF_4)] | Me, Pi | 32.5 kDa |
| TA17210 | Hypothetical protein |  | 65.7 kDa |
| TA17220 | Hypothetical protein (FAINT) |  | 62.3 kDa |
| TA06510 | p34 [[5](#_ENREF_5)] | MS | 36.9 kDa |
| TA10740 | Hypothetical protein |  | 38.8 kDa |
| TA13720 | Hypothetical protein |  | 176.6 kDa |
| TA18965 | putative udp-N-acetylglucosamine--dolichyl-phosphate n-acetylglucosaminephosphotransferase |  | 44.5 kDa |
| TA03755 | Sporozoite p67 surface antigen | Sp | 93.0 kDa |
| TA05320 | Hypothetical protein |  | 20.2 kDa |

1. Hertz-Fowler C, Peacock CS, Wood V, Aslett M, Kerhornou A, et al. (2004) GeneDB: a resource for prokaryotic and eukaryotic organisms. Nucleic Acids Res 32: D339-343.

2. Logan-Klumpler FJ, De Silva N, Boehme U, Rogers MB, Velarde G, et al. (2012) GeneDB--an annotation database for pathogens. Nucleic Acids Res 40: D98-108.

3. Pain A, Renauld H, Berriman M, Murphy L, Yeats CA, et al. (2005) Genome of the host-cell transforming parasite *Theileria annulata* compared with *T. parva*. Science 309: 131-133.

4. Glascodine J, Tetley L, Tait A, Brown D, Shiels B (1990) Developmental expression of a *Theileria annulata* merozoite surface antigen. Mol Biochem Parasitol 40: 105-112.

5. Xue G, von Schubert C, Hermann P, Peyer M, Maushagen R, et al. (2010) Characterisation of gp34, a GPI-anchored protein expressed by schizonts of *Theileria parva* and *T. annulata.* Mol Biochem Parasitol 172: 113-120.
